# Development of Rice Mutants with Enhanced Resilience to Drought Stress and Their Evaluation by Lab Assay, Field, and Multivariate Analysis

**DOI:** 10.1155/2024/4373987

**Published:** 2024-08-29

**Authors:** Shahwar Fatima, Muhammad Rashid, Amjad Hameed

**Affiliations:** Nuclear Institute for Agriculture and Biology College Pakistan Institute of Engineering and Applied Sciences (NIAB-C, PIEAS), Faisalabad, Pakistan

## Abstract

Drought is one of the foremost devastating abiotic stresses reported for rice crops. To improve the productivity of rice, diversity is being enlarged by induced mutation using a source of gamma rays. But this type of mutation rarely results in fruitful products because the chances of getting the desired mutant are very low. The present study aimed to evaluate the rice mutants against drought or osmotic stress. In this study, three experiments were conducted that comprised of seventy-one mutants originating from different doses of gamma rays (Cs^137^) along with parent RICF-160 and commercial variety (Kainat) were tested. In the first experiment, germination and seedling attributes were calculated under control and osmotic stress conditions created by using 16% (0.6 MPa) polyethylene glycol (PEG-6000). Results revealed that all the mutants exhibited significant (*p* < 0.01) responses to PEG-induced osmotic stress. Principal component biplot analysis (PCBA) revealed the first seventeen cumulative PCs with eigenvalues >1 contributed 88%. It was noted that the germination percentage (GP), germination rate (GR), coefficient velocity of germination (CVG), and seed vigor (SV) contributed maximum and positively in PC1. Results showed the highest germination percentage (GP) at 48 hrs in mutant NMSF-11 (88.9%) followed by NMSf-38 (73.3%). Similarly, the germination rate (GR) and coefficient velocity of germination (CVG) were measured highest in NMSF-11 (9.7 and 118.1%), respectively. In stress conditions, the mutants NMSF-35 and NMSF-36 depicted the highest GP, GR, and CVG. The maximum seed vigor (SV), shoot length (SL), root length (RL), and fresh weight (FW) were observed in mutants NMSF-50 and NMSF-51 under both conditions, whereas the mutants NMSF-59, NMSF-60, NMSF-64, and NMSF-67 showed lower values for SV, SL, RL, and FW. In the second experiment, a field trial was conducted at the Nuclear Institute for Agriculture and Biology (NIAB), Faisalabad, in two control and stress sets. A bit different trend was observed among all mutants for agronomic parameters under both conditions. In the third experiment, biochemical profiling was done in Marker Assisted Breeding (MAB) Lab-1, Plant Breeding and Genetics Division. A significant variation was seen in enzymatic antioxidants and chlorophyll content in both control and stress conditions. Under control conditions, the ascorbate peroxidase (APX) content was observed higher in mutant NMSF-49 (106.07 Units/g. f. wt.). In comparison with the stress, the ascorbate peroxidase activity was higher in NMSF-41 (82.34 Units/g. f. wt.). Catalase (CAT) activity was observed maximum in NMSF-29 (17.54 Units/g. f. wt.) and NMSF-40 (14.17 Units/g. f. wt.) under control and stress conditions, respectively. Peroxidase (POD) activity was observed maximum in NMSF-51 (22.55 Units/g. f. wt. and 10.84 Units/g. f. wt.) under control and stress conditions, respectively. In conclusion, to fit in the changing climate conditions for resilient rice crop production, the promising mutant lines may be used to transfer the desirable drought-tolerant/drought-resistant genes in rice germplasm.

## 1. Introduction

South Asia is the most sensitive to climate change among various agroecologies and has a large population with limited access to essential resources, such as water and arable land [1]. Most country priorities for improving drought tolerance in crops include increasing agricultural water productivity under rain-fed conditions [2, 3]. Plant growth and development are significantly hampered due to water shortage resulting in lower crop yields [4, 5]. Drought creates osmotic stress reactive oxygen species (ROS) in plant [6, 7] and animal [8, 9] cells that cause oxidative damage [10, 11] and is one of the reasons for gaps in world food production [2, 12, 13]. Drought tolerance can be achieved in two ways: by shortening the life cycle (escape) or by developing morphophysiological adaptations [14], such as root architecture, water use efficiency, and molecular changes to withstand the stress condition (true tolerance) [2, 15–17]. Drought obviously affects the crop productivity and hinders the seed germination [18–21]. Its main affect is on the growth and cell expansion [22, 23], which leads to a reduction in the shoot and root length in rice [5, 24, 25].

A recent estimate on climate change predicts that the intensity and frequency of drought will be of high magnitude in the future and will exhibit a significant impact on rice crop production [26, 27]. The drought stresses created by low rain-fed conditions highlighted the future scenario to have rice varieties suitable for the water stress conditions that may sustain rice crop production under variable climate conditions [28, 29].

To improve rice crops against water stresses, different mutation breeding techniques have been used successfully in rice. The mutagenesis in rice is advantageous due to its small genome, i.e., a small population is required to saturate the whole genome and to provide a larger allelic series for use in mutagenesis [30, 31]. Random mutations caused by physical and chemical agents have been applied to create genetic variability and for gene functional studies in rice [32]. In mutation breeding, instead of crossing, we just expose the high yielding cultivated variety to mutagen (physical and chemical) for improvement against one or other desirable genes without compromising the rice grain quality attributes [33]. This approach takes less time to uniform the breeding material and is non-GMO in practice. Induced mutation is the briefest conceivable strategy for the advancement of Basmati rice varieties/germplasm [34, 35]. Mutation breeding through gamma rays is a powerful and exceptionally fruitful methodology for the generation of commercial cultivars [34, 36]. The utilization of induced mutation in crop improvement has been demonstrated to be a successful way to deal with improved yield, quality, and protection from abiotic stresses [37, 38]. Induced mutation has been utilized in rice more than any other crop as confirmed by more than 443 rice mutant varieties listed in the FAO/IAEA Mutant Varieties Database [39, 40].

Rice (*Oryza sativa* L.), a monocot with shallow root architecture yet demand for high water for growth, is more susceptible to water shortage stress than other main food crops [41]. About 50% of the total populace (340 million individuals in South Asia and 140 million in Southeast Asia and sub-Saharan Africa) depends upon rice crop. Globally, Pakistan is known as one of the major rice-producing countries, where the area under rice crop is about 3.034 million hectares and contributes 3.1 percent of value added in agriculture and 0.6 percent in GDP (Pakistan Economic Survey, 2019−20).

To accelerate the breeding programs, screening strategies for drought resistance must be quick, cost-effective, and consistent for evaluating plant performance at the seedling stage [34, 36, 42]. Screening at seed and seedling stage is a reliable strategy and accelerates the process of screening stress-tolerant crops [43–47]. So the genetic association between germination and seedling traits will further help the breeders to improve drought tolerance in rice. Cluster analysis classifies drought-tolerant and drought-susceptible rice mutants for desirable traits based on genetic similarity. Thus, there is need to identify the suitable selection criteria that will enhance the efficiency of breeders to select drought-tolerant mutants from diverse populations. The current study was designed to evaluate diverse rice mutants developed through induced mutation, which have a higher tolerance to drought or osmotic stress in both lab and field conditions.

## 2. Results

### 2.1. PEG Experiment

#### 2.1.1. Germination Parameters

Under control conditions, results revealed maximum germination percentage at 48 hours in mutants NMSF-11 (88.9%) followed by NMSF-38 (73.3%), NMSF-2, and NMSF-5 (70.0%). The highest germination rate (GR) was also observed in NMSF-11 (9.7), which was followed by NMSF-38 (8.2) and NMSF-40 (8.1), whereas the lowest GR was observed in mutant NMSF-57 (1.3). Mutant NMSF-11 again showed the highest coefficient velocity of germination (CVG) (118.1) followed by 106.4 and 103.8 in NMSF-27 and NMSF-25, respectively. Similarly, as the GR, the minimum CVG (7.2) was also observed in mutant NMSF-57 (Supplementary Figures 1 and 2).

Drought stress adversely affects the germination. However, the highest germination percentage after two days was observed in NMSF-36 (50.4%) followed by NMSF-35 and NMSF-40, which were (46.7%) and (35.6%), respectively. The mutants NMSF-35 and NMSF-36 showed the maximum value of germination rate (GR) (6.4) followed by NMSF-40 (5.5). The minimum GR was observed in NMSF-60 and NMSF-56 that were (0.7) and (1.6), respectively. The mutants NMSF-35, NMSF-36, and NMSF-40 showed the highest values of CVG, which were 85.1, 71.8, and 71.7, respectively, whereas NMSF-64 and NMSF-60 showed lowest values for CVG, which were 0.5 and 0.8 (Supplementary Figure 3).

The analysis of variance of means showed that the variation among the mutants was highly significant (*p* ≤ 0.01) for all the parameters studied in both conditions (Table 1).

#### 2.1.2. Seedling Parameters

The current study showed a bit different trend in seedling parameters. Under control conditions, the higher seed vigor was observed in mutant NMSF-50 (23.8), whereas the lower value of seed vigor was seen in NMSF-59 (3.4). The shoot length was highest in NMSF-50 (13.5 cm) and NMSF-48 showed the lowest value for shoot length (4 cm). As for shoot length, the higher values for root length were recorded in mutants NMSF-51, NMSF-49, and NMSF-50, which were 12.8 cm, 11.3 cm, and 10.8 cm, respectively. NMSF-67 showed the lowest value for root length (1.9 cm). The value for fresh weight (FW) was maximum (0.947) in NMSF-50 and minimum (0.102) in NMSF-7. Dry weight (DW) was observed highest in NMSF-24 (0.176) and lowest in NMSF-59 and NMSF-67 (0.036). Similarly, in the stress condition, the mutant NMSF-51 showed higher seed vigor (20.4) and the lowest was observed in NMSF-60 (0.3). The maximum shoot length was observed in NMSF-51 (10.2 cm) and the minimum was observed in NMSF-64 (1.8 cm). The mutants NMSF-49 and NMSF-51 depict the highest value for root length (12.2 cm) and (10.2 cm), respectively, whereas the NMSF-60 showed the lowest value (0.8 cm). The higher fresh weight and dry weight in the stress condition were seen in NMSF-51 (0.367) and (0.098), respectively, whereas the mutants NMSF-4 and NMSF-40 showed the lowest values (0.046) and (0.022) for fresh weight and dry weight, respectively (Supplementary Figures 4–6).

### 2.2. Field Experiment

#### 2.2.1. Agronomic Parameters

Result showed highly significant differences in all the agronomic traits. The descriptive statistics of yield-related parameters under control conditions showed that NMSF-16 was tallest among all mutants and the mean values for plant height ranged from 113 cm to 147 nm. Similarly, the maximum productive tillers (31) in RICF-160 and the minimum (9) in NMSF-71 were observed. The highest value for panicle length was seen in NMSF-10 and NMSF-11 (35 cm), and the lowest was in NMSF-52 (25.3 cm). Total weight (59g) was calculated, which was higher in NMSF-22 and lower (20g) in NMSF-26. NMSF-19 depicted higher values for fertility percentage in control and stress conditions (95.2%) and (89.2%), respectively. Kainat showed lower values for fertility percentage (68%) and (47%) in both control and stress conditions, respectively. The maximum yield was observed in NMSF-18, which was 63.1 kg/ha, and the minimum 30 kg/ha was in NMSF-26. On the other hand, in the stress set, the plant height 138 cm was highest in NMSF-10 and the lowest 110 cm was observed in the commercial variety Kainat. The value for productive tillers was higher (26) in parent line RICF-160 and mutants NMSF-8 and NMSF-15, and the minimum value (9) in NMSF-54 and NMSF-56 was observed. The panicle length was maximum (32.5 cm) in NMSF-10 and minimum (22 cm) in NMSF-45. Mutant NMSF-11 depicted higher values for total weight (40.4 g) and yield (50 kg/ha). Lower values for total weight and yield were observed in NMSF-7 (17.5 g) and in NMSF-56 and NMSF-65 (22 kg/ha), respectively (Supplementary Figures 7–10) (Table 2). To validate the above-mentioned findings in different mutants, correlation between agronomic/yield-related parameters was determined (Table 3). In the control condition, productive tillers, total weight, and yield showed a negative association with plant height. Total spikelets and empty spikelets showed negative, whereas total weight, fertility, and yield showed positive association with productive tillers, and fertility and yield showed highly positive association with total weight in both sets. The analysis of variance (ANOVA) for mean squares indicated that highly significant differences were observed among all mutants for all the characters studied in both conditions as shown in Table 4.

### 2.3. Physio-Biochemical Profiling

A bit different trend was observed for physio-biochemical activities among all mutants under both control and stress conditions. A significant variation was seen in enzymatic antioxidants and chlorophyll content in both control and stress conditions. A significant decrease was also observed in all physio-biochemical assays among all mutants. Under control conditions, the ascorbate peroxidase (APX) content was observed higher (106.07 Units/g. f. wt.) in mutant NMSF-49 followed by NMSF-40 (105 Units/g. f. wt.). NMSF-6 depicted the lower content for APX (13.13 Units/g. f. wt.). NMSF-29 showed higher value (17.54 Units/g. f. wt.) and NMSF-20 showed lower value (4.64 Units/g. f. wt.) for catalase (CAT) activity. Peroxidase activity was higher in NMSF-36 (23.38 Units/g. f. wt.) followed by NMSF-51 (22.55 Units/g. f. wt.) and lower value was in NMSF-13 (2.49 Units/g. f. wt.). The chlorophyll content was maximum in NMSF-32 (51.133 *µ*g/g f. wt.) and minimum in NMSF-4 (11.5 *µ*g/g f. wt.). In comparison with the stress, the ascorbate peroxidase activity was higher in NMSF-41 and NMSF-40 (82.34 Units/g. f. wt.) (82.13 Units/g. f. wt.) and lower in NMSF-6 (11.78 Units/g. f. wt.). Maximum catalase activity was estimated in NMSF-40 (14.17 Units/g. f. wt.), and minimum was in NMSF-60 (2.31 Units/g. f. wt.). Peroxidase was measured maximum in NMSF-51 (10.84 Units/g. f. wt.) and minimum in NMSF-42 (0.21 Units/g. f. wt.). The chlorophyll content was measured higher in NMSF-14 (42.4 Units/g. f. wt.) followed by NMSF-44 (42.33 *µ*g/g f. wt.) and the lower value was seen in mutant NMSF-18 (6.33 *µ*g/g f. wt.). The analysis of variance and the mean squares are given in Supplementary Figures 11 and 12 (Table 5).

#### 2.3.1. Comparison for Yield and Yield Components

The plant height of mutants <10 cm and more was recorded in NMSF-26, NMSF-8, NMSF-25, NMSF-48, NMSF-24, NMSF-17, NMSF-1, NMSF-34, NMSF-46, NMSF-4, NMSF-36, NMSF-5, NMSF-22, NMSF-39, NMSF-2, NMSF-19, and NMSF-3. These mutants have 7.5% to 14.6% reduced height as compared to RICF-160 (132 cm) and control Kainat (133 cm). The mutant line NMSF-26 has maximum reduced height (114 cm) followed by NMSF-8 (115 cm) and NMSF-25 (116).

In the stress condition, the plant height of Kainat and RICF-160 was recorded as 126 cm and 127 cm, respectively. The mutants harboring plant height <126 were selected. The selected mutants are as follows: NMSF-1, NMSF-50, NMSF-68, NMSF-2, NMSF-3, NMSF-58, NMSF-5, NMSF-20, NMSF-51, NMSF-21, NMSF-62, NMSF-49, NMSF-4, NMSF-54, NMSF-53, NMSF-22, NMSF-10, NMSF-6, NMSF-52, NMSF-14, NMSF-55, NMSF-23, NMSF-38, NMSF-63, NMSF-44, NMSF-48, NMSF-27, NMSF-66, NMSF-35, NMSF-59, NMSF-15, NMSF-17, NMSF-30, NMSF-69, NMSF-42, NMSF-56, NMSF-33, NMSF-12, NMSF-37, NMSF-24, NMSF-31, NMSF-25, NMSF-64, NMSF-13, NMSF-61, and NMSF-57.

Productive tillers per plant in RICF-160 (16) and Kainat (18) were recorded. The mutants exhibited >18 tillers per plant were selected. The mutants NMSF-8, NMSF-15, NMSF-14, NMSF-20, NMSF-9, NMSF-19, NMSF-18, NMSF-24, and NMSF-5 with more tillers per plant were selected. The maximum tillers were recorded in NMSF-8 and NMSF-15 (26) followed by NMSF-14 and NMSF-20 (25), whereas NMSF-4 and NMSF-17 possess 19 tillers per plant.

In the stress condition, the RICF-160 and Kainat produced 11 and 12 tillers, respectively. The selected mutant lines that harbor >12 tillers were selected. The selected mutant lines (NMSF-15, NMSF-9, NMSF-1, NMSF-63, NMSF-4, NMSF-2, NMSF-10, NMSF-3, NMSF-65, NMSF-59, NMSF-8, NMSF-14, NMSF-58, NMSF-54, NMSF-25, NMSF-24, NMSF-11, NMSF-5, NMSF-70, NMSF-20, NMSF-66, NMSF-19, NMSF-28, NMSF-29, NMSF-13, NMSF-40, NMSF-62, NMSF-6, NMSF-17, NMSF-16, NMSF-32, NMSF-22, NMSF-61, NMSF-49, NMSF-30, NMSF-39, NMSF-67, NMSF-51, NMSF-31, NMSF-57, NMSF-60, NMSF-43, NMSF-21, NMSF-64, NMSF-44, NMSF-33, NMSF-55, NMSF-50, NMSF-47, NMSF-46, NMSF-34, NMSF-48, NMSF-18, NMSF-7, NMSF-69, NMSF-12, NMSF-35, NMSF-53, NMSF-37, NMSF-27, NMSF-36, and NMSF-26) have 12 to 28 tillers per plant.

Panicle length per plant in RICF-160 (30) and Kainat (28) was recorded. The mutant lines NMSF-11, NMSF-9, NMSF-44, NMSF-16, NMSF-17, NMSF-54, NMSF-37, NMSF-14, NMSF-57, NMSF-58, NMSF-34, NMSF-71, NMSF-18, NMSF-60, NMSF-63, NMSF-22, and NMSF-35 exhibited 31 cm to 35 cm panicle length. The maximum panicle length was recorded in NMSF-11 (35 cm) followed by NMSF-9 and NMSF-44 (33 cm).

In the stress condition, mutants with >29 cm panicle length were selected. The selected mutants (NMSF-10, NMSF-58, NMSF-44, NMSF-9, NMSF-60, NMSF-39, NMSF-67, NMSF-65, NMSF-28, and NMSF-11) ranged from 30 to 33 cm panicle length.

Regarding total spikelets per panicle, the RICF-160 and Kainat have 212 and 148 spikelets per panicle, respectively. The mutant lines NMSF-23, NMSF-11, NMSF-25, NMSF-22, NMSF-31, NMSF-29, NMSF-37, NMSF-58, and NMSF-44 have 219 to 255 spikelets per panicle. The maximum spikelets per panicle were recorded in NMSF-23 and NMSF-11 (255) followed by NMSF-25 (251).

In the stress condition, RICF-160 and Kainat have 182 and 133 spikelets per panicle. The selected mutants, namely, NMSF-67, NMSF-24, NMSF-49, NMSF-44, NMSF-46, NMSF-13, NMSF-30, NMSF-40, NMSF-31, NMSF-22, NMSF-50, NMSF-70, NMSF-43, NMSF-47, NMSF-60, NMSF-28, NMSF-34, NMSF-25, NMSF-69, NMSF-33, NMSF-71, NMSF-36, and NMSF-58, have >182 spikelets per panicle. The spikelets per panicle ranged from 184 to 232. Maximum spikelets per panicle were recorded in NMSF-67 (232) followed by NMSF-24 (227) and NMSF-49 (220).

Regarding yield per plant (gm), it was noted that all the mutant lines are higher in yield over the Kainat (18 gm), whereas mutant lines NMSF-20, NMSF-71, NMSF-65, NMSF-68, NMSF-26, NMSF-10, NMSF-69, and NMSF-56 were lower in yield than that in the RICF-160 (35 gm). Other mutant lines yield from 36 to 83 gm per plant. The maximum yield was recorded in NMSF-22 (83 gm), NMSF-9 (76 gm), and NMSF-14 (70 gm).

In the stress condition, the yield per plant in RICF-160 (31 gm) and Kainat (34 gm) was recorded. The selected mutants that have >34 gm were selected. The range of yield per plant in selected mutants (NMSF-9, NMSF-11, NMSF-4, NMSF-14, NMSF-15, NMSF-63, NMSF-25, NMSF-2, NMSF-48, NMSF-13, NMSF-17, NMSF-58, NMSF-57, NMSF-3, NMSF-39, NMSF-29, NMSF-47, NMSF-49, NMSF-1, NMSF-62, NMSF-5, NMSF-30, NMSF-64, NMSF-20, NMSF-28, NMSF-6, NMSF-37, NMSF-67, NMSF-32, NMSF-24, NMSF-46, NMSF-50, NMSF-40, NMSF-8, NMSF-31, NMSF-45, NMSF-51, NMSF-21, NMSF-59, NMSF-44, NMSF-19, NMSF-18, NMSF-53, and NMSF-42) ranged from 35 gm to 65 gm.

The fertility percentage of mutants NMSF-19, NMSF-2, NMSF-3, NMSF-67, NMSF-1, NMSF-18, NMSF-32, NMSF-9, NMSF-15, NMSF-16, NMSF-20, NMSF-54, NMSF-49, NMSF-6, NMSF-37, NMSF-4, NMSF-64, NMSF-69, NMSF-17, NMSF-14, NMSF-38, NMSF-5, NMSF-41, NMSF-13, NMSF-63, NMSF-12, NMSF-7, NMSF-46, NMSF-36, NMSF-31, NMSF-35, NMSF-44, NMSF-56, NMSF-40, NMSF-68, NMSF-65, NMSF-57, NMSF-21, NMSF-29, NMSF-22, NMSF-66, NMSF-71, NMSF-28, NMSF-53, NMSF-25, NMSF-23, NMSF-45, NMSF-33, NMSF-42, NMSF-43, and NMSF-11 ranged from 78 to 95%, whereas the RICF-160 and Kainat possessed 78 and 66%, respectively.

In the stress condition, for fertility percentage, it was noted that Kainat and RICF-160 exhibited 80 and 78%. The mutant lines (NMSF-54, NMSF-63, NMSF-59, NMSF-20, NMSF-19, NMSF-14, NMSF-16, NMSF-4, NMSF-60, NMSF-15, NMSF-18, NMSF-62, NMSF-58, NMSF-66, NMSF-1, NMSF-2, NMSF-3, NMSF-64, NMSF-17, NMSF-53, NMSF-61, NMSF-65, NMSF-5 NMSF-11, NMSF-9, and NMSF-43) have more than 82% fertility percentage. The maximum fertility was recorded in NMSF-54 (91%) followed by NMSF-63 (91%) and NMSF-59 (90%).

The mutants NMSF-22, NMSF-9, NMSF-14, NMSF-13, NMSF-18, NMSF-2, NMSF-17, NMSF-23, NMSF-25, NMSF-24, NMSF-21, NMSF-29, NMSF-31, NMSF-12, NMSF-47, NMSF-15, NMSF-42, NMSF-3, NMSF-41, NMSF-44, NMSF-30, NMSF-19, NMSF-11, NMSF-64, NMSF-5, NMSF-35, NMSF-48, NMSF-8, NMSF-34, NMSF-4, NMSF-58, NMSF-52, NMSF-45, NMSF-46, NMSF-49, NMSF-62, NMSF-1, NMSF-50, NMSF-57, NMSF-51, NMSF-6, NMSF-39, NMSF-16, NMSF-7, NMSF-38, and NMSF-59 with total weight of 28 gm to 59 gm were selected in the RICF-160 (27 gm) and Kainat (16 gm).

In the stress condition, the total weight (24 gm) was recorded in both the RICF-160 and Kainat. The selected mutant lines (NMSF-58, NMSF-15, NMSF-11, NMSF-65, NMSF-4, NMSF-14, NMSF-25, NMSF-63, NMSF-2, NMSF-64, NMSF-6, NMSF-57, NMSF-9, NMSF-13, NMSF-17, NMSF-66, NMSF-54, NMSF-3, NMSF-5, NMSF-29, NMSF-47, NMSF-49, NMSF-1, NMSF-70, NMSF-62, NMSF-30, NMSF-20, NMSF-60, NMSF-61, NMSF-28, NMSF-37, NMSF-67, NMSF-32, NMSF-24, NMSF-46, NMSF-45, NMSF-40, NMSF-50, NMSF-10, NMSF-8, NMSF-21, NMSF-31, NMSF-39, NMSF-51, NMSF-59, NMSF-55, NMSF-42, NMSF-44, NMSF-19, NMSF-18, NMSF-53, NMSF-48, and NMSF-71) have TW, which ranged from 24 gm to 57 gm.

#### 2.3.2. Comparison for Physio-Biochemical Traits

Stomatal conductance (SC) of Kainat (125.4) and RICF-160 (141) was recorded. The mutants exhibiting < 125 values were selected. Among these selected mutants (NMSF-16, NMSF-18, NMSF-9, NMSF-21, NMSF-53, NMSF-42, NMSF-56, NMSF-23, NMSF-19, NMSF-45, NMSF-48, NMSF-55, NMSF-12, NMSF-22, and NMSF-13), NMSF-16 exhibited 7.3 SC followed by NMSF-18 (80.4) and NMSF-9 (89.1). These mutants have low stomatal opening, which may be useful for stress tolerance.

In the stress condition, the SC values less than RIVF-160 (55) and Kainat (65) were recorded. The mutants, namely, NMSF-16, NMS,F-53, NMSF-12, NMSF-57, NMSF-18, NMSF-19, NMSF-23, NMSF-56, NMSF-17, NMSF-9, NMSF-45, NMSF-13, NMSF-3, NMSF-22, NMSF-42, NMSF-70, NMSF-7, NMSF-4, NMSF-21, NMSF-48, NMSF-10, NMSF-20, NMSF-1, NMSF-5, and NMSF-71, have 22 to 54.7 SC values. The least SC value under stress was recorded in NMSF-16 (22.0) followed by NMSF-53 (25.7) and NMSF-12 (26).

Regarding transpiration rate (TR), the mutants, namely, NMSF-16, NMSF-17, NMSF-18, NMSF-3, NMSF-6, NMSF-2, NMSF-4, NMSF-13, NMSF-1, NMSF-70 and NMSF-61, with less transpiration rate were selected. Minimum transpiration rate in NMSF-16 (39.3) followed by NMSF-17 (40.3) and NMSF-18 (41.1) was recorded.

In the stress set, the TR of RICF-160 was 16.7 and Kainat (19.7). The mutants having TR less than RICF-160 were NMSF-16, NMSF-5, NMSF-45, NMSF-4, NMSF-69, NMSF-55, NMSF-23, NMSF-6, NMSF-42, NMSF-56, NMSF-61, NMSF-71, NMSF-65, NMSF-57, NMSF-21, NMSF-13, NMSF-68, NMSF-48, NMSF-2, NMSF-10, NMSF-20, NMSF-3, NMSF-67, NMSF-70, NMSF-1, NMSF-12, and NMSF-19.

Mutants (NMSF-16, NMSF-9, NMSF-3, NMSF-4, NMSF-19, NMSF-10, and NMSF-13) with low photosynthetic rates were selected. The NMSF-16 has 1.4, NMSF-9 has 1.5, and NMSF-3 has 1.6 photosynthetic rates, whereas the RICF-160 has 1.7 and Kainat has 1.9 photosynthetic rates (PRs).

In the stress condition, the PRs of mutants NMSF-4, NMSF-69, NMSF-3, NMSF-60, NMSF-61, NMSF-56, NMSF-71, and NMSF-65 were found low with range of 0.2 to 0.4, whereas the RICF-160 and Kainat were 0.4.

Regarding ascorbate peroxidase (APX), the RICF-160 (44.1) and Kainat (44.5) were recorded. Except NMSF-45, NMSF-66, NMSF-55, NMSF-5, NMSF-4, and NMSF-6, all other mutant lines exhibited higher values of APX. The selected 65 mutant lines exhibited higher values of APX than the RICF-160 and Kainat. The maximum values were recorded in NMSF-49 (106.1), NMSF-40 (105.0), and NMSF-51 (102.0).

In the stress condition, 70 mutant lines exhibited higher values of APX except one line, i.e., NMSF-11, which exhibited 11.8 APX value, whereas in stress, the Kainat and RICF have 33.6 and 33.2 APX values, respectively. The lines higher in APX are as follows: NMSF-41, NMSF-40, NMSF-50, NMSF-39, NMSF-11, NMSF-14, NMSF-49, NMSF-15, NMSF-28, NMSF-51, NMSF-29, NMSF-8, NMSF-27, NMSF-52, NMSF-46, NMSF-54, NMSF-44, NMSF-43, NMSF-38, NMSF-25, NMSF-36, NMSF-35, NMSF-33, NMSF-26, NMSF-32, NMSF-37, NMSF-31, NMSF-34, NMSF-24, NMSF-12, NMSF-67, NMSF-63, NMSF-2, NMSF-58, NMSF-62, NMSF-18, NMSF-17, NMSF-65, NMSF-64, NMSF-20, NMSF-19, NMSF-59, NMSF-22, NMSF-21, NMSF-3, NMSF-1, NMSF-4, NMSF-7, NMSF-57, NMSF-30, NMSF-9, NMSF-47, NMSF-10, NMSF-16, NMSF-53, NMSF-48, NMSF-13, NMSF-56, NMSF-68, NMSF-55, NMSF-71, NMSF-69, NMSF-23, NMSF-45, NMSF-60, NMSF-42, NMSF-66, NMSF-5, NMSF-61, and NMSF-70.

The CAT values in Kainat (5.6) and RICF-160 (5.6) were recorded. The NMSF-12, NMSF-56, NMSF-5, NMSF-48, NMSF-19, NMSF-7, NMSF-57, NMSF-23, NMSF-22, NMSF-18, NMSF-16, NMSF-42, NMSF-61, NMSF-6, NMSF-17, NMSF-21, NMSF-45, NMSF-2, NMSF-30, NMSF-60, and NMSF-20 exhibited less values for CAT activity. Forty-eight mutants have higher CAT values, which range from 5.7 to 17.5. The maximum CAT activity was recorded in NMSF-29 (17.5) followed by NMSF-40 (5.9) and NMSF-26 (5.3).

In the stress condition, the CAT values of Kainat (3.3) and RICF-160 (3.8) were recorded. Among the genotypes, 62 were selected under the stress condition. The CAT values of selected lines ranged from 3.8 to 14.2. The selected lines were designated as follows: NMSF-40, NMSF-36, NMSF-49, NMSF-31, NMSF-35, NMSF-39, NMSF-50, NMSF-32, NMSF-26, NMSF-33, NMSF-28, NMSF-41, NMSF-29, NMSF-27, NMSF-24, NMSF-15, NMSF-52, NMSF-51, NMSF-6, NMSF-25, NMSF-14, NMSF-8, NMSF-47, NMSF-38, NMSF-10, NMSF-62, NMSF-37, NMSF-64, NMSF-44, NMSF-46, NMSF-54, NMSF-65, NMSF-63, NMSF-3, NMSF-13, NMSF-67, NMSF-1, NMSF-9, NMSF-7, NMSF-58, NMSF-11, NMSF-59, NMSF-70, NMSF-66, NMSF-43, NMSF-4, NMSF-19, NMSF-53, NMSF-68, NMSF-23, NMSF-34, NMSF-18, NMSF-22, NMSF-12, NMSF-16, NMSF-71, NMSF-61, NMSF-56, NMSF-55, NMSF-5, NMSF-69, and NMSF-42.

In the controlled condition for POD activity, the selected 50 mutant lines have POD values from 6.8 to 23.4. The maximum POD activity was recorded in NMSF-36 (23.4) followed by NMSF-51 (22.5) and NMSF-40 (22.4). The mutant lines exhibiting values less that RICF-160 and Kainat were NMSF-30, NMSF-69, NMSF-71, NMSF-60, NMSF-48, NMSF-3, NMSF-56, NMSF-6, NMSF-4, NMSF-5, NMSF-2, NMSF-9, NMSF-42, and NMSF-13.

In the stress condition, 36 mutant lines exhibited higher values of POD over Kainat (4.5), which ranged from 4.9 to 10.8. The maximum value was recorded in NMSF-51 (10.8), NMSF-50 (10.5), and NMSF-33 (9.8). When compared with RICF-160 (3.1), besides the mentioned 36 mutant lines, further 18 lines also exhibited higher values of POD. The minimum POD value of the selected mutant NMSF-30 was 3.2.

The mutant lines exhibited good response under the stress condition than both the Kainat and RICF-160 were as follows: NMSF-51, NMSF-50, NMSF-33, NMSF-52, NMSF-40, NMSF-35, NMSF-36, NMSF-38, NMSF-39, NMSF-41, NMSF-37, NMSF-49, NMSF-34, NMSF-47, NMSF-31, NMSF-29, NMSF-44, NMSF-32, NMSF-24, NMSF-43, NMSF-28, NMSF-26, NMSF-11, NMSF-27, NMSF-63, NMSF-62, NMSF-15, NMSF-25, NMSF-14, NMSF-58, NMSF-54, NMSF-46, NMSF-59, NMSF-67, and NMSF-8.

The chlorophyll contents in mutants (NMSF-32, NMSF-14, NMSF-44, NMSF-46, NMSF-40, NMSF-31, NMSF-34, NMSF-38, and NMSF-36) ranged from 42.9 to 51.1, whereas in Kainat, 42.9 were recorded. When compared with RICF-160, besides above mutants, the mutants NMSF-33, NMSF-43, NMSF-41, NMSF-6, NMSF-67, NMSF-50, NMSF-68, NMSF-60, NMSF-58, NMSF-63, NMSF-52, NMSF-1, NMSF-71, NMSF-51, NMSF-37, NMSF-54, NMSF-28, NMSF-70, NMSF-64, NMSF-29, and NMSF-59 ranged from 39.7 to 42.7, whereas the RICF-160 had chlorophyll value as 39.5.

In the stress condition, the mutant lines NMSF-14, NMSF-44, NMSF-46, NMSF-32, and NMSF-40 have 41.5 to 42. 2 chlorophyll contents, whereas the Kainat have 41.1 chlorophyll contents. When compared with RICF-160 except the above-mentioned lines, the mutant lines NMSF-58, NMSF-43, NMSF-59, NMSF-52, NMSF-50, NMSF-6, NMSF-60, NMSF-54, NMSF-41, NMSF-51, NMSF-36, NMSF-67, NMSF-61, NMSF-38, NMSF-63, NMSF-30, NMSF-66, NMSF-37, NMSF-49, NMSF-47, NMSF-69, NMSF-34, NMSF-45, NMSF-70, NMSF-15, NMSF-42, NMSF-29, NMSF-3, NMSF-56, NMSF-31, NMSF-64, NMSF-8, NMSF-71, NMSF-22, NMSF-62, NMSF-13, NMSF-65, NMSF-57, NMSF-25, NMSF-68, NMSF-1, and NMSF-2 have higher values than the RICF-160 (33.7).

It is concluded that from the comparison study, out of 71, 39 mutant lines were selected. These mutant lines, namely, NMSF-3, NMSF-58, NMSF-1, NMSF-4, NMSF-13, NMSF-67, NMSF-5, NMSF-15, NMSF-25, NMSF-31, NMSF-44, NMSF-49, NMSF-50, NMSF-59, NMSF-61, NMSF-62, NMSF-63, NMSF-65, NMSF-2, NMSF-9, NMSF-10, NMSF-11, NMSF-14, NMSF-19, NMSF-20, NMSF-24 NMSF-28 NMSF-37, NMSF-42, NMSF-47, NMSF-51, NMSF-53, NMSF-54, NMSF-57, NMSF-60, NMSF-64, NMSF-69, NMSF-70, and NMSF-71, were selected for rice breeding program against the stress environmental conditions.

### 2.4. Cluster Analysis

**Figure 1:**
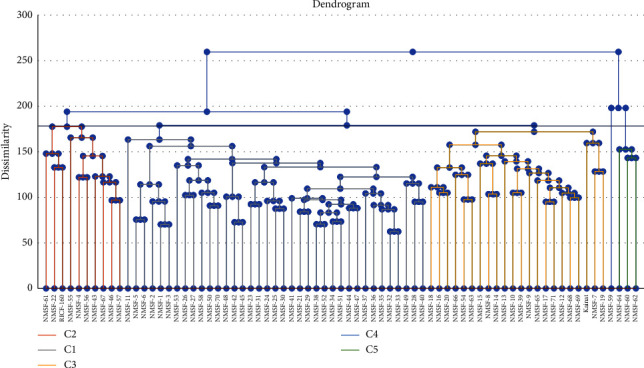
Tree diagram based on germination, seedling, yield, and physio-biochemical traits for different rice mutants under control and drought stress conditions.

For all mutants, a tree diagram was constructed through the hierarchical cluster analysis by using all the germination, seedling, yield, quality, and biochemical data under both control and water stress conditions. Cluster analysis categorized seventy-one mutants along with their parent line RICF-160 and commercial variety Kainat into five clusters as shown in Table 6 and Figure 1. Cluster I contained maximum (37) number of mutants. Ten mutants were grouped in cluster II. Twenty-two were grouped in cluster III. Mutant NMSF-59 was placed into cluster IV, while three mutants NMSF-60, NMSF-62, and NMSF-64 were grouped in cluster V.

#### 2.4.1. Correlation Analysis

**Figure 2:**
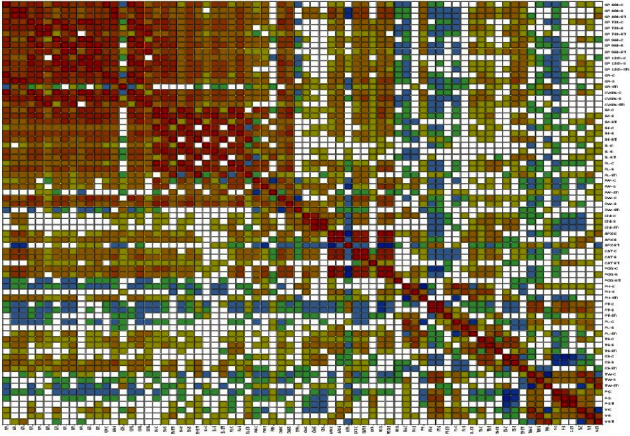
Correlation matrix showing Pearson's correlation among germination, seedling, yield, and physio-biochemical traits for different rice mutants under control and drought stress conditions.

Correlation (Pearson) for all growth, seedling, yield, and physio-biochemical traits under both control and drought stress was carried [48] out using XLSTAT 2023, with 95% confidence interval (Figure 2 and Supplementary Table 2). Values in the bold form are different from 0 with a significance level alpha = 0.05. Values with negative sign showed a negative correlation.

Under the control condition, germination percentage (GP%) at 48 hours was positively correlated with germination percentage at 72, 96, and 120 hours, germination rate (GR), coefficient velocity of germination (CVG), seed vigor (SV), dry weight (DW), and empty spikelets (ES) in both conditions, while with peroxidase (POD) and total spikelets (TS) only in the control condition and in the stress condition, it was positively correlated with seedling height (SH), shoot length (SL), root length (RL), and fresh weight (FW). Germination percentage at 48 hours negatively correlated with the fertility percentage (F %) in control condition. Germination rate in both control and stress conditions was positively correlated with all the parameters except seedling height (SH), shoot length (SL), root length (RL), fresh weight (FW), ascorbate peroxidase (APX), and empty spikelets (ES) and also no correlation with chlorophyll (Chl) content, productive tillers (PT), panicle length (PL), total spikelets (TS), total weight (TW), and yield (Y) in both control and stress conditions. It was negatively correlated with plant height (PH) and fertility (F %) in the control condition.

Shoot length under the control condition showed a positive correlation with seed vigor (SV), seedling height, root length, fresh weight, and dry weight in both conditions, while with germination percentage at 72 hours only in control and germination percentages at 96 and 120 hours and coefficient velocity of germination (CVG) only in stress condition. It was negatively correlated with productive tillers (PT) and total weight in control condition and showed no correlation with other parameters. There was no correlation between shoot length in the stress condition and fresh weight, chlorophyll content, ascorbate peroxidase activity (APX), catalase activity (CAT), peroxidase (POD), plant height, productive tillers, panicle length, total spikelets, empty spikelets, total weight, fertility, and yield. Results showed that there was no association between root length in both conditions and panicle length, total spikelets, empty spikelets, total weight, fertility, and yield while negatively correlated with productive tillers.

Plant height under the control condition showed no correlation with all parameters except panicle length while negatively correlated with germination percentage at 48, 72, and 96 hours, germination rate, coefficient velocity of germination, dry weight and the stress tolerance index of the plant height, panicle length, and empty spikelets. On the other hand, under the stress condition, plant height positively correlates with panicle length, total spikelets, fresh weight, ascorbate peroxidase, and peroxidase in both conditions. It was noted that productive tillers under the stress condition showed positive correlation with total weight, fertility, and yield in both conditions, while negatively correlated with root length, seedling height, seed vigor, and ascorbate peroxidase activity in control and germination percentage at 48, 72, 96 hours, germination rate, and coefficient velocity of germination in stress condition. Empty spikelets, peroxidase activity, catalase activity, chlorophyll content (Chl), and fresh weight (FW) in both conditions showed a negative correlation with productive tillers under stress conditions.

Results revealed yield under control conditions showed a positive correlation with germination percentage at 72 and 96 hours, coefficient velocity of germination in control condition, stress tolerance index (STI) of ascorbate peroxidase, productive tillers, panicle length, fertility, and total weight in both conditions, while negatively correlated with chlorophyll content in stress, empty spikelets in control, and STI of total weight. Among physio-biochemical parameters, chlorophyll content positively correlates with germination percentage at 48 hours, germination rate, total spikelets, empty spikelets, POD activity, CAT activity, root length, fresh weight, and dry weight, under both conditions. Ascorbate peroxidase activity under the control condition showed a positive association with germination percentage at 48 hours, germination rate, coefficient velocity of germination, seed vigor, root length, fresh weight, plant height, total spikelets in stress conditions, and in both conditions with dry weight, catalase activity, and peroxidase activity, while negatively correlates with productive tillers and total weight.

#### 2.4.2. Principal Component Biplot Analysis

For a better understanding of the relationship among mutants and to extract the important and useful information present in the data matrix, the principal component analysis was performed [48] for all mutants and 24 traits with their stress tolerance index under both control and stress conditions. It also minimized the number of traits that describes the maximum percentage of variability present in the data. The eigenvalue is very crucial that decides which principal components are important and useful for further study. The highest eigenvalue reflected as the best representative of system with the aspects of principal components [49]. Under both conditions, 17 principal components (PCs) depicted more than 1.0 eigenvalue and explained 88.343% variability. The first five components were most persuasive: PC-I contributed 25.861% of total variability; PC-II, PC-III, PC-IV, and PC-V individually contributed 10.745%, 6.955%, 6.468%, and 6.028%, respectively, while the cumulatively PC-I, PC-II, PC-III, PC-IV, and PC-V contributed 25.861%, 36.606%, 43.561%, 50.029%, and 56.057% respectively, of the total variability (Supplementary Table 1).

**Figure 3:**
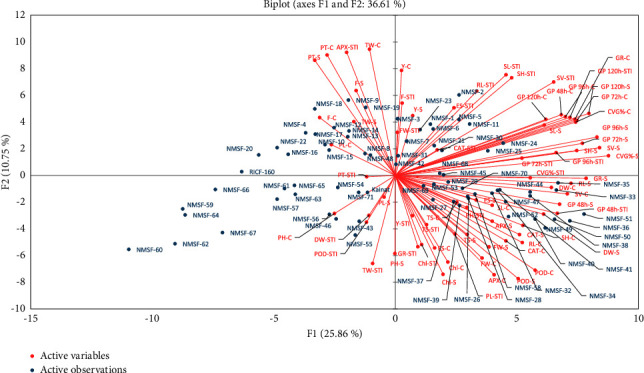
Biplots of first two principal components (PC-I and PC-II) for control and stress conditions.

**Figure 4:**
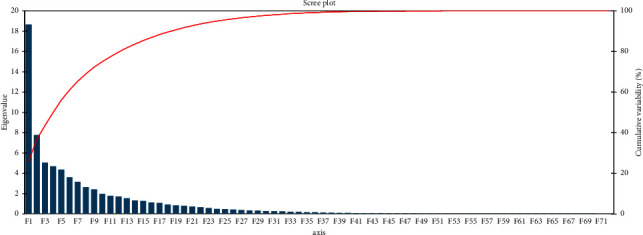
Scree plot representing cumulative variability and eigenvalues for studied parameters.

Out of 24 traits under both conditions, 22 parameters showed positive factor loading in PC-I, while germination percentage (at 48, 72, 96, and 120 hours), germination rate, coefficient velocity of germination, seed vigor, and seedling height of both conditions (control and stress) and stress tolerance index (STI) had the greatest effect in PC-I. In PC-II, 16 parameters exhibited the positive factor loading with productive tillers, total weight, shoot length, root length, fertility, and yield having the greatest effect (Supplementary Table 1). For more authentic identification of mutants with maximum values for one or more traits, mutant-by-trait biplot was constructed against PC-I and PC-II for all the mutants and all the traits under both conditions (Figure 3). It explained the trait description of a mutant [50]. Vector line was drawn from the origin of the biplot to understand the interrelation between mutants and traits. Genotypic performance that differs one mutant from the others can be assessed by the distance of the mutant from the origin of the biplot. The more distant mutants could have more values for one or more traits. A scree plot showed cumulative variability and eigenvalues for studied parameters (Figure 4).

## 3. Discussion

In the present scenario of climate change, drought is one of the most severe abiotic stresses hampering seed germination, plant growth, and crop production [51–53]. A high rate and consistency of germination under water stress conditions is very much important for good crop growth to achieve good productivity [54]. Among different methods of breeding, mutation breeding has gained popularity among researchers due to its utilization in plant biotechnology because of certain limitations of different methods of breeding like hybridization and transgenic techniques [55]. Among different mutagens, physical mutagens (gamma rays) are widely used due to their easy handling as compared to chemical mutagens, which may cause carcinogenic effects [56]. Due to the irradiation of seeds, reactive oxygen species (ROS) or free radicals are generated in cells and cause random mutations [57]. To create genetic variability in the desired variety for the trait of interest, the seeds of the parent varieties RICF-160 and RICF-159 and RICF-152 were exposed to different doses of gamma rays [58]. In this study, we examined and matched the difference among mutant lines in control and water stress conditions [59].

In the present study, the first experiment was conducted on 71 rice mutants along with their parent line RICF-160 and commercial variety Kainat was subjected to PEG-induced drought stress for the estimation of germination and seedling growth. Eight mutants were created from 200 Gy dose, eleven from 250 Gy dose, and five from 300 Gy dose of RICF-160. Two mutants were created from 200 Gy dose, one form 250 Gy dose and two from 300 Gy dose of RICF-159. Forty-two mutants were created from 300 Gy dose of RICF-152 (Table 7). During the last few decades, mutagenesis has resurfaced in plant breeding. Plant mutagenesis, which produces new variety in crop plants, combined with in vitro selection and plant biotechnology methods enables breeders to select for characters that were previously difficult to achieve in breeding program.

Different germination and seedling parameters were observed and recorded. The present results highlighted significant differences among the mutants exposed to drought stress with a remarkable improved germination [60–63]. These results are consistent with those of other studies that have reported that high concentrations of PEG reduce the final germination percentages of lentils [64–66]. The seedling height was observed more over the control in mutants NMSF-2, NMSF-5, NMSF-14, NMSF-23, NMSF-24, NMSF-30, NMSF-33, NMSF-35, NMSF-48, and NMSF-59.

Shoot length was observed more in mutants NMSF-50, NMSF-51, and NMSF-52 in both conditions. Similarly, the value of root length and seed vigor was observed highest in the mutants NMSF-49, NMSF-50, and NMSF-51. Similar findings were reported by the earlier researchers on lentils [67, 68]. These findings suggest that seed germination and root length, fresh weight, dry weight, and seedling vigor can be used as traits for the rapid selection of drought-tolerant cultivars. The ability to develop extensive root systems contributes to differences among cultivars for drought tolerance, and root length is considered an important trait in the selection of drought-resistant cultivars [69, 70]. Thus, root morphology and/or growth rate may be instrumental to select drought-tolerant varieties [71–73]. The root length reduction in different mutants under drought stress may be associated with a reduced cellular division and elongation during germination [74, 75]. The mutants NMSF-32, NMSF-33, NMSF-34, NMSF-35, NMSF-36, NMSF-38, NMSF-40, NMSF-41, NMSF-44, NMSF-47, NMSF-49, NMSF-50, NMSF-51, and NMSF-52 were away from the center and have approved the effect of mutation breeding for creation of genetic variability. Therefore, these mutants can be considered valuable drought-tolerant germplasm.

Oxidative stress is regarded as a major damaging factor in plants [76] and animals [9, 77] exposed to a variety of abiotic stresses including drought [78]. So, it is important to examine the relationship between the imposition of drought stress and the induction of oxidative stress in the partial halophytic crop rice [79–82]. APX appears to constitute a basic mechanism of deployment for antioxidative defense in plants [47, 83]. Our results indicated a decline in CAT activity under drought stress, which suggests, at least here, that CAT appears not to be an effective scavenger of H_2_O_2_ in our case [84]. Such observations suggest that increased water deficit induced severe oxidative stress in rice plants, where antioxidant defense system seemingly fails to combat the oxidative damage [85].

## 4. Material and Methods

### 4.1. Lab Experiment

#### 4.1.1. Experimental Design and Treatments

The experiment was carried out at the Plant Breeding and Genetics Division, Nuclear Institute for Agriculture and Biology (NIAB), Pakistan. A completely randomized design (CRD) under factorial arrangement was used in this study consisting of two sets: drought stress (16% Polyethylene glycol 6000 that could create the osmotic pressure of −0.6 MPa) and control set (autoclaved distilled water) with three replications for each set in a completely controlled environment suitable for rice crop germination.

#### 4.1.2. Plant Culture and Harvest

**Figure 5:**
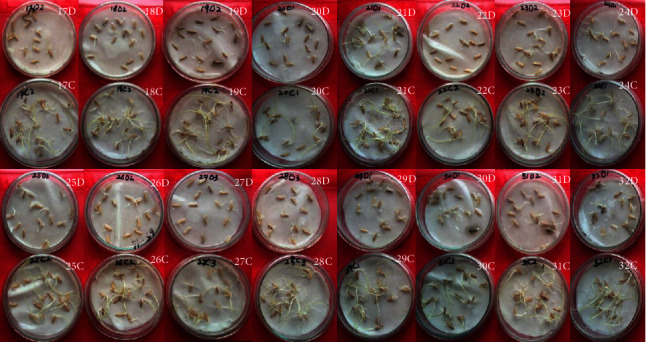
Seed germination after 15 days in both control “*C*” and drought “*D*” conditions.

Seventy-one rice mutants originated at NIAB from different doses of gamma rays (137Cs) were analyzed for drought response along with parent variety RICF-160 and commercial variety (Kainat). To conduct this experiment, healthy and uniform seeds were selected. Selected seeds were treated with Topsin M to avoid any contamination. About 15 seeds of each mutant were placed on autoclaved Petri plates containing two layers of Whatman No. 1 filter paper as shown in Figure 5. Sterilized PEG solution and distilled water were used as growth medium for stressed and control sets, respectively. Seeds in each Petri plates were placed at sufficient distance to allow the optimum growth of shoot and root length. Each plate was irrigated with 1 mL of autoclaved water (in case of control) and 1 mL of 16% PEG-6000 (in case of stress) with a pipette on a daily basis to moisten the filter paper, and the plates were placed in the dark to allow germination at 37°C [86, 87]. The experiment was continued for 12 days from the day of seed sowing for the control set and 15 days for the stress set as the water stress-induced late germination of seeds. After 15 days, the experiment was harvested for data recording.

#### 4.1.3. Germination Attributes

The data for germination and germination-related parameters were collected on a daily basis. The data were recorded for different germination parameters, namely, germination percentage (GP) and coefficient of velocity of germination (CVG), which give the indication of the rapidity of germination, and germination rate (GR) basically gives an idea of the percentage of seeds germinating per day.

#### 4.1.4. Seedling Attributes

Three seedlings were selected randomly from each replicate for the data collection of growth parameters such as root length (RL), shoot length (SL), seedling height (SH), seed vigor (SV), fresh weight (FW), and dry weight (DW) following the earlier worker [43]. The seedlings with 2 mm of RL were measured as germinated [88]. The root length, shoot length, and seedling height were measured in cm by using a scale. Fresh weight was measured using an analytical balance. The seedlings were then oven-dried at 70°C for 24 h for the estimation of the dry weight.

### 4.2. Field Trial

#### 4.2.1. Experimental Design and Treatments

The same seventy-one mutants along with parent line RICF-160 and commercial variety Kainat were used in the field experiment. The experiment was conducted in the experimental field area at the Nuclear Institute for Agriculture and Biology, Faisalabad. Two water regiments were used and irrigated and water stress conditions using randomized complete block design with three replicates for both conditions. Forty-day-old seedlings were transplanted 20 cm apart between rows and 15 cm within the rows. Irrigation was stopped for one of the sets at the initiation of booting stage to create the drought stress and last till maturity. All necessary precautions were taken to maintain uniform plant population in each treatment per replication.

#### 4.2.2. Plant Culture and Harvest

All the recommended practices were followed along with the plant protection measure to raise a good crop. Observations were recorded, and the data were subjected to statistical analysis. Then, the samples were collected at flag leaf stage in labeled zipper bags from both normal and stressed sets for antioxidant estimation. The samples were then stored at −20°C to ensure the preserve integrity. The analysis was carried out at Marker Assisted Breeding (MAB) Lab-1, Plant Breeding and Genetics Division, Nuclear Institute for Agriculture and Biology (NIAB), Faisalabad, Pakistan.

#### 4.2.3. Agronomic Parameters

The data for different agronomic traits, namely, plant height (cm), productive tillers, panicle length (cm), total spikelet, empty spikelet, fertility (% age), total weight (g), and yield (kg per hectare), were recorded. Finally, quantitative data were subjected to ANOVA using Statistix 8.1.

#### 4.2.4. Physiological and Biochemical Parameters

*(1) Chlorophyll Content*. Chlorophyll content was measured as SPAD (soil plant analysis development) value from the third upper leaf of the three plants [89].

*(2) Antioxidant Enzymes*. Fresh leaves (1 g) were ground in 1.5 ml (50 mM) potassium phosphate buffer (pH 7.4). Samples were then centrifuged at 14000 rpm for 10 minutes at 4°C. The supernatant was separated and used for the determination of the different enzymatic and nonenzymatic activities. All the data were taken in triplicate. The extract was used for the assay of the following antioxidant activities as described earlier [48].

*(3) Ascorbate Peroxidase (APX) Activity*. APX activity was measured by using 50 *μ*l sample extract, 1000 *μ*l H_2_O_2_, and assay buffer (10 mM ascorbic acid 3.4 ml, 500 mM EDTA 10 ml, 200 mM potassium phosphate buffer 25 ml, and distilled water 50 ml) 1000 *μ*l [90]. The reaction was initiated by the addition of 1 ml of 10% (v/v) H_2_O_2_, and the oxidation rate of ascorbic acid was estimated by the following of the decrease in absorbance at 290 nm for 1 min [91].

*(4) Catalase (CAT) Activity*. CAT was estimated by the following method described by Beers and Sizer [92]. For the measurement of CAT activity, assay solution contained 50 mM phosphate buffer (pH 7.4) 2 ml, 59 mM H_2_O_2_, and 100 *μ*l and enzyme extract 100 *μ*l. The decrease in the absorbance of the reaction solution at 240 nm was recorded after every 20s for 1 minute.

*(5) Peroxidase (POD) Activity*. Activity of POD was measured using the method of Chance and Maehly [93] with some modification. For the measurement of POD activity, assay solution contained 535 *μ*l distilled water, 20 mM guaiacol 100 *μ*l, 400 mM H_2_O_2_ 100 *μ*l, 200 mM phosphate buffer (pH 7.0), and 250 *μ*l and 15 *μ*l enzyme extracts. The reaction was initiated by adding the enzyme extract. The increase in the absorbance of the reaction solution at 470 nm was recorded after every 20 s for 1 minute.

*(6) Statistical Analysis*. The screening experiments were conducted in three repeats using a randomized completely block design (RCBD) [48]. The significance was determined by ANOVA and Tukey (HSD) test at *p* < 0.05 by using XLSTAT 2023 software. To check the response of the mutants under control and stress (drought) treatments, bar graphs were constructed based on mean ± S.E. In graphs, bars with different alphabets were significantly different from each other. Principle component analysis (PCA) for all the parameters under both conditions was performed, and for the first two principal components (PC-I and PC-II), biplots were constructed by using the same software. Correlation (Pearson test) and cluster analysis were also performed by algometric hierarchical clustering for all mutants under all traits by using the same software.

## 5. Conclusion

It is concluded that out of 71, 14 mutants (NMSF-32, NMSF-33, NMSF-34, NMSF-35, NMSF-36, NMSF-38, NMSF-40, NMSF-41, NMSF-44, NMSF-47, NMSF-49, NMSF-50, NMSF-51, and NMSF-52), which were found in +ve *X*/*Y*-axis of ordination, were desirable and other 57 have the potential to give better response to drought stress. It was concluded that to fit in the changing climate conditions for resilient rice crop production, the promising mutant lines may be used to transfer the desirable drought-tolerant/drought-resistant genes in rice germplasm.

## Figures and Tables

**Table 1:** Mean squares from analysis of variance under control and stressed condition.

Characters	Control		Stress	
	Mutants	Error	Mutants	Error
GP at 48 hr	1534.66^∗∗^	0.75	556.84^∗∗^	0.6
GP at 72 hr	1842.55^∗∗^	0.97	1501^∗∗^	0.36
GP at 96 hr	1006.66^∗∗^	0.55	1684.11^∗∗^	0.36
GP at 120 hr	757.75^∗∗^	0.78	1334.02^∗∗^	0.5
GR	11.48^∗∗^	0.05	4.98^∗∗^	0.03
CVG	2377.66^∗∗^	2.1	1348.49^∗∗^	0.96
SL	11.45^∗∗^	0.15	9.55^∗∗^	0.17
RL	19.57^∗∗^	0.13	14.76^∗∗^	0.14
SH	47.33^∗∗^	0.19	38.81^∗∗^	0.13
SV	59.99^∗∗^	0.29	50.68^∗∗^	0.24
FW	0.14^∗∗^	0	0.02^∗∗^	0
DW	0.0022^∗∗^	0	0.0017^∗∗^	0

GP = germination percentage; GR = germination rate; CVG = coefficient velocity of germination; SL = shoot length; RL = root length; SH = seedling height; SV = seed vigor; FW = fresh weight; DW = dry weight. ^∗∗^highly significant at *p* < 0.01.

**Table 2:** Descriptive statistics of yield attributes of rice mutants.

Traits	Minimum		Maximum		Mean		SE		CV (%)	
	Control	Stress	Control	Stress	Control	Stress	Control	Stress	Control	Stress
Plant height (cm)	113	110	147	138	128.01	125.03	0.574	0.755	0.78	1.05
Productive tillers	9	9	31	26	15.76	16.19	0.632	1.234	6.95	13.21
Panicle length (cm)	32.5	22	35	25.5	29.058	27.548	0.838	0.783	5	4.92
Total spikelets	130	107	255	227	177.32	165.85	7.076	11.819	6.91	12.34
Empty spikelets	6	11	116	66	34.834	37.379	5.023	7.249	24.98	33.59
Total weight (g)	20	17.5	59	40.4	31.506	27.782	2.268	1.351	12.47	8.42
Fertility (%)	68	47	95.2	89.2	80.586	77.958	2.727	3.976	5.86	8.83
Yield (kg/ha)	30	22	63.1	50	45.74	36.53	2.794	1.427	10.58	6.77

**Table 3:** Pair-wise correlation between yield attributes of mutants.

Traits			PH	PT	PL	TS	ES	TW	F
PT	C	−0.156^∗^						
	S	−0.103ns						

PL	C	0.115ns	0.072ns					
	S	0.109ns	0.179^∗∗^					

TS	C	−0.074ns	−0.226^∗∗^	0.243^∗∗^				
	S	0.074ns	−0.198^∗∗^	0.314^∗∗^				

ES	C	0.006ns	−0.183^∗∗^	−0.177^∗∗^	0.461^∗∗^			
	S	0.139^∗^	−0.346^∗∗^	−0.013ns	0.624^∗∗^			

TW	C	−0.221^∗∗^	0.434^∗∗^	0.249^∗∗^	0.415^∗∗^	−0.132ns		
	S	−0.149^∗^	0.574^∗∗^	0.347^∗∗^	−0.03ns	−0.298^∗∗^		

F	C	0.063ns	0.138^∗^	0.277^∗∗^	−0.127ns	−0.928^∗∗^	0.335^∗∗^	
	S	−0.152^∗^	0.375^∗∗^	0.155^∗^	−0.253^∗∗^	−0.897^∗∗^	0.363^∗∗^	

Yield	C	−0.185^∗∗^	0.385^∗∗^	0.313^∗∗^	0.431^∗∗^	−0.064ns	0.925^∗∗^	0.258^∗∗^
	S	−0.094ns	0.49^∗∗^	0.178^∗∗^	−0.034ns	−0.193^∗∗^	0.568^∗∗^	0.241^∗∗^

C = control; S = stress; PH = plant height (cm); PT = productive tillers; PL = panicle length (cm); TS = total spikelets; ES = empty spikelets; TW = total weight (g); F = fertility (%);ns = nonsignificant. ^∗∗^significant at *p* ≤ 0.01, ^∗^significant at *p* ≤ 0.05.

**Table 4:** Mean square of yield attributes of both (control and stress) sets.

SOV	DF		PH (cm)		PT		PL (cm)		TS	
	C	S	C	S	C	S	C	S	C	S
Replication	2	2	7.288	29.409	4.59	3.436	5.06	0.045	12.61	73.68
Mutants	172	172	148.337^∗∗^	77.733^∗∗^	48.343^∗∗^	45.539^∗∗^	11.343^∗∗^	7.592^∗∗^	3501.16^∗∗^	3032.24^∗∗^
Error	144	144	0.987	1.708	1.198	4.571	2.108	1.837	150.21	419.05

SOV	DF		ES		TW (g)		F (%)		Yield (kg/ha)	
	C	S	C	S	C	S	C	S	C	S

Replication	2	2	78.226	221.332	25.093	4.59	24.068	65.248	20.358	5.069
Mutants	172	172	825.734^∗∗^	641.707^∗∗^	264.448^∗∗^	107.773^∗∗^	206.005^∗∗^	140.388^∗∗^	387.074^∗∗^	205.148^∗∗^
Error	144	144	75.688	157.64	15.506	5.477	22.312	47.417	23.415	6.107

SOV = source of variance; DF = degree of freedom; C = control; S = stress; PH = plant height (cm); PT = productive tillers; PL = panicle length (cm); TS = total spikelets; ES = empty spikelets; TW = total weight (g); F = fertility (%). ^∗∗^significant at *p* ≤ 0.01.

**Table 5:** ANOVA and mean square for physio-biochemical traits of both (control and stress) sets.

SOV	DF		APX		CAT		POD		Chlorophyll	
	C	S	C	S	C	S	C	S	C	S
Replication	2	2	22.28	4.21	0.38	0.03	3.44	0.26	1.73	4.39
Mutants	172	172	1528.02^∗∗^	468.82^∗∗^	32.99^∗∗^	15.71^∗∗^	118.32^∗∗^	25.25^∗∗^	73.53^∗∗^	144.84^∗∗^
Error	144	144	13.48	3.14	0.16	0.06	1.35	0.12	2.94	3.24

SOV = source of variance; DF = degree of freedom; C = control; S = stress; APX = ascorbate peroxidase; CAT = catalase; POD = peroxidase. ^∗∗^significant at alpha = 0.05.

**Table 6:** Different mutants grouped into five clusters along with their parent line RICF-160 and commercial variety Kainat.

Cluster	Mutants
I	NMSF-1, NMSF-2, NMSF-3, NMSF-5, NMSF-6, NMSF-11, NMSF-21, NMSF-23, NMSF-24, NMSF-25, NMSF-26, NMSF-27, NMSF-28, NMSF-29, NMSF-30, NMSF-31, NMSF-32, NMSF-33, NMSF-34, NMSF-35, NMSF-36, NMSF-37, NMSF-38, NMSF-40, NMSF-41, NMSF-42, NMSF-44, NMSF-45, NMSF-47, NMSF-48, NMSF-49, NMSF-50, NMSF-51, NMSF-52, NMSF-53, NMSF-58, and NMSF-70
II	NMSF-4, NMSF-22, NMSF-43, NMSF-46, NMSF-55, NMSF-56, NMSF-57, NMSF-61, NMSF-67, and RICF-160
III	NMSF-7, NMSF-8, NMSF-9, NMSF-10. NMSF-12, NMSF-13, NMSF-14, NMSF-15, NMSF-16, NMSF-17, NMSF-18, NMSF-19, NMSF-20, NMSF-39, NMSF-54, NMSF-63, NMSF-65, NMSF-66, NMSF-68, NMSF-69, NMSF-71, and Kainat
IV	NMSF-59
V	NMSF-60, NMSF-62, and NMSF-64

**Table 7:** Details of the experimental material along with their parent line and commercial variety (CV) and gamma radiation doses from which these mutants were originated.

Mutants	Dose (Gy)	Parent
NMSF-1	200	RICF-160
NMSF-2	200	RICF-160
NMSF-3	200	RICF-160
NMSF-4	200	RICF-160
NMSF-5	200	RICF-160
NMSF-6	200	RICF-160
NMSF-7	200	RICF-160
NMSF-8	200	RICF-160
NMSF-9	250	RICF-160
NMSF-10	250	RICF-160
NMSF-11	250	RICF-160
NMSF-12	250	RICF-160
NMSF-13	250	RICF-160
NMSF-14	250	RICF-160
NMSF-15	250	RICF-160
NMSF-16	250	RICF-160
NMSF-17	250	RICF-160
NMSF-18	250	RICF-160
NMSF-19	250	RICF-160
NMSF-20	300	RICF-160
NMSF-21	300	RICF-160
NMSF-22	300	RICF-160
NMSF-23	300	RICF-160
NMSF-24	300	RICF-160
NMSF-25	200	RICF-159
NMSF-26	200	RICF-159
NMSF-27	250	RICF-159
NMSF-28	300	RICF-159
NMSF-29	300	RICF-159
NMSF-30	300	RICF-152
NMSF-31	300	RICF-152
NMSF-32	300	RICF-152
NMSF-33	300	RICF-152
NMSF-34	300	RICF-152
NMSF-35	300	RICF-152
NMSF-36	300	RICF-152
NMAF-37	300	RICF-152
NMSF-38	300	RICF-152
NMSF-39	300	RICF-152
NMSF-40	300	RICF-152
NMSF-41	300	RICF-152
NMSF-42	300	RICF-152
NMSF-43	300	RICF-152
NMSF-44	300	RICF-152
NMSF-45	300	RICF-152
NMSF-46	300	RICF-152
NMSF-47	300	RICF-152
NMSF-48	300	RICF-152
NMSF-49	300	RICF-152
NMSF-50	300	RICF-152
NMSF-51	300	RICF-152
NMSF-52	300	RICF-152
NMSF-53	300	RICF-152
NMSF-54	300	RICF-152
NMSF-55	300	RICF-152
NMSF-56	300	RICF-152
NMSF-57	300	RICF-152
NMSF-58	300	RICF-152
NMSF-59	300	RICF-152
NMSF-60	300	RICF-152
NMSF-61	300	RICF-152
NMSF-62	300	RICF-152
NMSF-63	300	RICF-152
NMSF-64	300	RICF-152
NMSF-65	300	RICF-152
NMSF-66	300	RICF-152
NMSF-67	300	RICF-152
NMSF-68	300	RICF-152
NMSF-69	300	RICF-152
NMSF-70	300	RICF-152
NMSF-71	300	RICF-152
RICF-160		Parent line
Kainat		CV

## Data Availability

All data are included in the manuscript in the form of tables and graphs.

## Supplementary Materials

Supplementary Materials Graphs are presented in Figure S1 to S12. Table S1: principal component analysis for growth, seedling, morphophysiological, yield-related, and biochemical parameters under control and drought stress conditions. Table S2: correlation matrix (Pearson).

## References

[1] Somasundaram J., Sinha N., Dalal R. C. (2020). No-till farming and conservation agriculture in South Asia–issues, challenges, prospects and benefits. *Critical Reviews in Plant Sciences*.

[2] Alam G. M., Shrestha R. B. (2021). Promote sustainability of family farming for climate-resilient food systems in South Asia. *Regional Action Plan to Implement the UNDFF for Achieving the SDGs in South Asia*.

[3] Mabhaudhi T., Nhamo L., Mpandeli S. (2021). Enhancing crop water productivity under increasing water scarcity in South Africa. *Climate Change Science*.

[4] Raza A., Razzaq A., Mehmood S. S. (2019). Impact of climate change on crops adaptation and strategies to tackle its outcome: a review. *Plants*.

[5] Yadav T., Kumar A., Yadav R., Yadav G., Kumar R., Kushwaha M. (2020). Salicylic acid and thiourea mitigate the salinity and drought stress on physiological traits governing yield in pearl millet-wheat. *Saudi Journal of Biological Sciences*.

[6] Sarker U., Oba S. (2018). Catalase, superoxide dismutase and ascorbate-glutathione cycle enzymes confer drought tolerance of Amaranthus tricolor. *Scientific Reports*.

[7] Sarker U., Hossain M. N., Iqbal M. A., Oba S. (2020). Bioactive components and radical scavenging activity in selected advance lines of salt-tolerant vegetable amaranth. *Frontiers in Nutrition*.

[8] Yue S., Qian J., Du J. (2023). Heat stress negatively influence mammary blood flow, mammary uptake of amino acids and milk amino acids profile of lactating holstein dairy cows. *Pakistan Veterinary Journal*.

[9] Al-Saeed F. A., Naz S., Saeed M. H. (2023). Oxidative stress, antioxidant enzymes, genotoxicity and histopathological profile in *Oreochromis niloticus* exposed to lufenuron. *Pakistan Veterinary Journal*.

[10] Achten W. M., Maes W., Reubens B. (2010). Biomass production and allocation in Jatropha curcas L. seedlings under different levels of drought stress. *Biomass and Bioenergy*.

[11] Sarker U., Oba S., Daramy M. (2020). Nutrients, minerals, antioxidant pigments and phytochemicals, and antioxidant capacity of the leaves of stem amaranth. *Scientific Reports*.

[12] Ali A., Chaudhary F. M. (2008). Germination behavior of wheat (*Triticum aestivum*) varieties to artificial ageing under varying temperature and humidity. *Pakistan Journal of Botany*.

[13] Almuzaini A. M. (2023). Flow of zoonotic toxoplasmosis in food chain. *Pakistan Veterinary Journal*.

[14] Sarker U., Oba S. (2018). Augmentation of leaf color parameters, pigments, vitamins, phenolic acids, flavonoids and antioxidant activity in selected Amaranthus tricolor under salinity stress. *Scientific Reports*.

[15] Sarker U., Islam M. T., Oba S. (2018). Salinity stress accelerates nutrients, dietary fiber, minerals, phytochemicals and antioxidant activity in Amaranthus tricolor leaves. *PLoS One*.

[16] Rady M. M., Belal H. E., Gadallah F. M., Semida W. M. (2020). Selenium application in two methods promotes drought tolerance in Solanum lycopersicum plant by inducing the antioxidant defense system. *Scientia Horticulturae*.

[17] Azizullah A., Taimur N., Häder D.-P. (2021). Novel biotechnological substances in higher plants. *Natural Bioactive Compounds*.

[18] HongBo S., ZongSuo L., MingAn S. (2006). Osmotic regulation of 10 wheat (*Triticum aestivum* L.) genotypes at soil water deficits. *Colloids and Surfaces B: Biointerfaces*.

[19] Akhter J., Sabir S. A., Lateef Z., Ashraf M. Y., Haq M. A. (2008). Relationships between carbon isotope discrimination and grain yield, water-use efficiency and growth parameters in wheat (*Triticum aestivum* L.) under different water regimes. *Pakistan Journal of Botany*.

[20] Zaman Q. U., Aslam Z., Yaseen M. (2018). Zinc biofortification in rice: leveraging agriculture to moderate hidden hunger in developing countries. *Archives of Agronomy and Soil Science*.

[21] Zaman Q., Aslam Z., Rashid M., Khaliq A., Yaseen M. (2018). Influence of zinc fertilization on morpho-physiological attributes, growth, productivity and hematic appraisal of paddy rice. *JAPS: Journal of Animal and Plant Sciences*.

[22] Kusaka M., Ohta M., Fujimura T. (2005). Contribution of inorganic components to osmotic adjustment and leaf folding for drought tolerance in pearl millet. *Physiologia Plantarum*.

[23] Farooq M., Wahid A., Kobayashi N., Fujita D., Basra S. (2009). Plant drought stress: effects, mechanisms and management. *Sustainable Agriculture*.

[24] Sikuku P., Onyango J., Onyango J. (2012). Physiological and biochemical responses of five nerica rice varieties (*Oryza sativa* L.) to water deficit at vegetative and reproductive stage. *Agriculture and Biology Journal of North America*.

[25] Zaman Q., Aslam Z., Rashid M. (2020). Zinc nutrition application augments mor-pho-physiological attributes, productivity and grain zinc bioavailability of Paddy Rice. *Journal of Applied Botany and Food Quality*.

[26] Bates B., Kundzewicz Z., Wu S. (2008). *Climate Change and Water*.

[27] Wassmann R., Jagadish S., Sumfleth K. (2009). Regional vulnerability of climate change impacts on Asian rice production and scope for adaptation. *Advances in Agronomy*.

[28] Sarker U., Oba S. (2019). Protein, dietary fiber, minerals, antioxidant pigments and phytochemicals, and antioxidant activity in selected red morph Amaranthus leafy vegetable. *PLoS One*.

[29] Mackill D. J., Ismail A. M., Pamplona A. M., Sanchez D. L., Carandang J. J., Septiningsih E. M. (2010). Stress tolerant rice varieties for adaptation to a changing climate. *Crop, Environment and Bioinformatics*.

[30] Potupureddi G., Balija V., Ballichatla S. (2021). Mutation resource of Samba Mahsuri revealed the presence of high extent of variations among key traits for rice improvement. *PLoS One*.

[31] Luqman T., Qamar Z.-u., Tabasum A. (2023). Genetic characterization of coarse and basmati rice (Oryza sativa L.) through microsatellite markers and morpho-agronomic traits. *Genetic Resources and Crop Evolution*.

[32] Awan F. S., Sadia B., Altaf J., Habib M., Hameed K., Hussain S. (2021). Genetic variability through induced mutation. *Genetic Variation*.

[33] Saxena K., Bohra A., Choudhary A. K. (2021). The alternative breeding approaches for improving yield gains and stress response in pigeonpea (Cajanus cajan). *Plant Breeding*.

[34] Himes A., Emerson P., McClung R., Renninger H., Rosenstiel T., Stanton B. (2021). Leaf traits indicative of drought resistance in hybrid poplar. *Agricultural Water Management*.

[35] Rashid M., Cheema A. A., Ashraf M. (2008). Numerical analysis of variation among basmati rice mutants. *Pakistan Journal of Botany*.

[36] Soe H., Myat M., Khaing Z., Nyo N., Phyu P. (2016). Development of drought tolerant mutant from rice var. Manawthukha through mutation breeding technique using 60Co gamma source. *International Journal of Innovative Research*.

[37] Bibi S., Khan I. A., Bughio H., Odhano I. A., Asad M. A., Khatri A. (2009). Genetic differentiation of rice mutants based on morphological traits and molecular marker (RAPD). *Pakistan Journal of Botany*.

[38] Jayakodi M., Padmarasu S., Haberer G. (2020). The barley pan-genome reveals the hidden legacy of mutation breeding. *Nature*.

[39] Lal R. K., Chanotiya C. S., Gupta P. (2020). Induced mutation breeding for qualitative and quantitative traits and varietal development in medicinal and aromatic crops at CSIR-CIMAP, Lucknow (India): past and recent accomplishment. *International Journal of Radiation Biology*.

[40] Ahloowalia B., Maluszynski M., Nichterlein K. (2004). Global impact of mutation-derived varieties. *Euphytica*.

[41] Zaman Q. U., Rashid M., Nawaz R. (2021). Silicon fertilization: a step towards cadmium-free fragrant rice. *Plants*.

[42] Hameed A., Goher M., Iqbal N. (2010). Evaluation of seedling survivability and growth response as selection criteria for breeding drought tolerance in wheat. *Cereal Research Communications*.

[43] Al-Mudaris M. (1998). Notes on various parameters recording the speed of seed germination. *Der Tropenlandwirt-Journal of Agriculture in the Tropics and Subtropics*.

[44] Panthuwan G., Fokai S., Cooper M., Rajatasereekul S., O’Toole J. (2002). Yield response of rice genotypes to different types of drought under rainfed lowlands. Part 1: grain yield and yield components. *Field Crops Research*.

[45] Li W., Zhang H., Zeng Y. (2020). A salt tolerance evaluation method for sunflower (Helianthus annuus L.) at the seed germination stage. *Scientific Reports*.

[46] Cai K., Chen X., Han Z. (2020). Screening of worldwide barley collection for drought tolerance: the assessment of various physiological measures as the selection criteria. *Frontiers in Plant Science*.

[47] Zafar S. A., Hameed A., Ashraf M. (2020). Agronomic, physiological and molecular characterisation of rice mutants revealed the key role of reactive oxygen species and catalase in high-temperature stress tolerance. *Functional Plant Biology*.

[48] Jameel S., Hameed A., Shah T. M. (2021). Investigation of distinctive morpho-physio and biochemical alterations in desi chickpea at seedling stage under irrigation, heat, and combined stress. *Frontiers in Plant Science*.

[49] Kumar A., Kumar A., Ranjan R., Kumar S., Rajani K., Singh P. (2019). Principal component analysis of agro-morpho-genetic traits in desi chickpea (Cicer arietinum L.).

[50] Turner N. C. (2017). Turgor maintenance by osmotic adjustment, an adaptive mechanism for coping with plant water deficits. *Plant, Cell and Environment*.

[51] Aslam M., Ahmad K., Maqbool M. A., Bano S., Zaman Q. U., Talha G. M. (2014). Assessment of adaptability in genetically diverse chickpea genotypes (Cicer arietinum L.) based on different physio-morphological standards under ascochyta blight inoculation. *International Journal of Advanced Research*.

[52] Aslam M., Maqbool M. A., Zaman Q. U., Latif M., Ahmad R. (2013). Responses of mungbean genotypes to drought stress at early growth stages. *International Journal of Basic and Applied Sciences*.

[53] Seleiman M. F., Al-Suhaibani N., Ali N. (2021). Drought stress impacts on plants and different approaches to alleviate its adverse effects. *Plants*.

[54] Chao S., Mitchell J., Fukai S. (2021). Factors determining genotypic variation in the speed of rice germination. *Agronomy*.

[55] Yousuf M., Alim D. (2020). Selection and hybridization techniques for stress management and quality improvement in rice. *Rice Research for Quality Improvement: Genomics and Genetic Engineering*.

[56] Melsen K., van de Wouw M., Contreras R. (2021). Mutation breeding in ornamentals. *HortScience*.

[57] Sharma P., Jha A. B., Dubey R. S., Pessarakli M. (2012). Reactive oxygen species, oxidative damage, and antioxidative defense mechanism in plants under stressful conditions. *Journal of Botany*.

[58] Zhao H., Xi C., Tian M. (2020). Identification of potential radiation responsive metabolic biomarkers in plasma of rats exposed to different doses of cobalt-60 gamma rays. *Dose-Response*.

[59] Kim S. L., Kim N., Lee H. (2020). High-throughput phenotyping platform for analyzing drought tolerance in rice. *Planta*.

[60] Ahmad S., Ahmad R., Ashraf M., Ejaz A., Waraich A. (2009). Sunflower (Helianthus Annuus L.) response to drought stress at germination and seedling growth stages. *Pakistan Journal of Botany*.

[61] Muscolo A., Sidari M., Anastasi U., Santonoceto C., Maggio A. (2014). Effect of PEG-induced drought stress on seed germination of four lentil genotypes. *Journal of Plant Interactions*.

[62] Radić V., Balalić I., Jaćimović G., Nastasić A., Savić J., Marjanović-Jeromela A. (2019). Impact of drought and salt stress on seed germination and seedling growth of maize hybrids. *Genetika*.

[63] Kouighat M., Hanine H., El Fechtali M., Nabloussi A. (2021). First report of sesame mutants tolerant to severe drought stress during germination and early seedling growth stages. *Plants*.

[64] Siahsar B. A., Ganjali S., Allahdoo M. (2010). Evaluation of drought tolerance indices and their relationship with grain yield of lentil lines in drought-stressed and irrigated environments. *Australian Journal of Basic and Applied Sciences*.

[65] Jamaati-e-Somarin S., Zabihi-e-Mahmoodabad R. (2011). Evaloation of droght tolerance indices of lentil varietes. *Advances in Environmental Biology*.

[66] Reza Yousefi A., Rashidi S., Moradi P., Mastinu A. (2020). Germination and seedling growth responses of Zygophyllum fabago, Salsola kali L. and Atriplex canescens to PEG-induced drought stress. *Environments*.

[67] Wu C., Wang Q., Xie B., Wang Z., Cui J., Hu T. (2011). Effects of drought and salt stress on seed germination of three leguminous species. *African Journal of Biotechnology*.

[68] Chiraz C. H., Afef H. N., Donia B., Houda G. (2014). Variations in *α*-*β*-amylase and *α*-glycosidase activities in two genotypes of wheat under NaCl salinity stress. *Plant Breeding and Seed Science*.

[69] Abd Allah A., Badawy S. A., Zayed B., El-Gohary A. (2010). The role of root system traits in the drought tolerance of rice (Oryza sativa L.). *Journal of Plant Production*.

[70] Vicente M. J., Martínez-Díaz E., Martínez-Sánchez J. J., Franco J. A., Bañón S., Conesa E. (2020). Effect of light, temperature, and salinity and drought stresses on seed germination of Hypericum ericoides, a wild plant with ornamental potential. *Scientia Horticulturae*.

[71] Wahbi A., Gregory P. (1995). Growth and development of young roots of barley (Hordeum vulgare L.) genotypes. *Annals of Botany*.

[72] Malik A. I., Colmer T. D., Lambers H., Setter T. L., Schortemeyer M. (2002). Short‐term waterlogging has long‐term effects on the growth and physiology of wheat. *New Phytologist*.

[73] Bernau V. M., Jardon Barbolla L., McHale L. K., Mercer K. L. (2020). Germination response of diverse wild and landrace Chile peppers (Capsicum spp.) under drought stress simulated with polyethylene glycol. *PLoS One*.

[74] Fraser T. E., Silk W. K., Rost T. L. (1990). Effects of low water potential on cortical cell length in growing regions of maize roots. *Plant Physiology*.

[75] Basal O., Szabó A., Veres S. (2020). PEG-induced drought stress effects on soybean germination parameters. *Journal of Plant Nutrition*.

[76] Demidchik V. (2012). Reactive oxygen species and oxidative stress in plants. *Plant Stress Physiology*.

[77] Akpinar D., Mercan T., Demir H., Ozdemir S., Demir C., Kavak S. (2023). Protective effects of thymoquinone on doxorubicin-induced lipid peroxidation and antioxidant enzyme levels in rat peripheral tissues. *Pakistan Veterinary Journal*.

[78] Nguyen G. N., Hailstones D. L., Wilkes M., Sutton B. G. (2009). Drought-induced oxidative conditions in rice anthers leading to a programmed cell death and pollen abortion. *Journal of Agronomy and Crop Science*.

[79] Sgherri C. L., Pinzino C., Navari-Izzo F. (1996). Sunflower seedlings subjected to increasing stress by water deficit: changes in O2.- production related to the composition of thylakoid membranes. *Physiologia Plantarum*.

[80] Hameed A., Goher M., Iqbal N. (2013). Drought induced programmed cell death and associated changes in antioxidants, proteases, and lipid peroxidation in wheat leaves. *Biologia Plantarum*.

[81] Zahid Z., Khan M. K. R., Hameed A. (2021). Dissection of drought tolerance in upland cotton through morpho-physiological and biochemical traits at seedling stage. *Frontiers in Plant Science*.

[82] Shah T. M., Imran M., Atta B. M. (2020). Selection and screening of drought tolerant high yielding chickpea genotypes based on physio-biochemical indices and multi-environmental yield trials. *BMC Plant Biology*.

[83] Madhusudhan R., Ishikawa T., Sawa Y., Shigeoka S., Shibata H. (2003). Characterization of an ascorbate peroxidase in plastids of tobacco BY‐2 cells. *Physiologia Plantarum*.

[84] Hertwig B., Streb P., Feierabend J. r. (1992). Light dependence of catalase synthesis and degradation in leaves and the influence of interfering stress conditions. *Plant Physiology*.

[85] Sharma P., Dubey R. S. (2005). Drought induces oxidative stress and enhances the activities of antioxidant enzymes in growing rice seedlings. *Plant Growth Regulation*.

[86] Abdul Q., Abdul R., Muhammad A., Matthew A. J. (2011). Water stress causes differential effects on germination indices, total soluble sugar and proline content in wheat (*Triticum aestivum* L.) genotypes. *African Journal of Biotechnology*.

[87] Badr A., El-Shazly H. H., Tarawneh R. A., Börner A. (2020). Screening for drought tolerance in maize (Zea mays L.) germplasm using germination and seedling traits under simulated drought conditions. *Plants*.

[88] Afzal I., Aslam N., Mahmood F., Hameed A., Irfan S., Ahmad G. (2004). Enhancement of germination and emergence of canola seeds by different priming techniques.

[89] Ramesh K., Chandrasekaran B., Balasubramanian T., Bangarusamy U., Sivasamy R., Sankaran N. (2002). Chlorophyll dynamics in rice (Oryza sativa) before and after flowering based on SPAD (chlorophyll) meter monitoring and its relation with grain yield. *Journal of Agronomy and Crop Science*.

[90] Dixit V., Pandey V., Shyam R. (2001). Differential antioxidative responses to cadmium in roots and leaves of pea (Pisum sativum L. cv. Azad). *Journal of Experimental Botany*.

[91] Chen G.-X., Asada K. (1989). Ascorbate peroxidase in tea leaves: occurrence of two isozymes and the differences in their enzymatic and molecular properties. *Plant and Cell Physiology*.

[92] Beers R. F., Sizer I. W. (1952). A spectrophotometric method for measuring the breakdown of hydrogen peroxide by catalase. *Journal of Biological Chemistry*.

[93] Chance B., Maehly A. (1955). Assay of catalases and peroxidases. *Methods in Enzimology*.

