# Supplementary material for: Development of Rice Mutants with Enhanced Resilience to Drought Stress and Their Evaluation by Lab Assay, Field, and Multivariate Analysis
Source: Scientifica (Cairo). 2024 Aug 29;2024:4373987. doi: 10.1155/2024/4373987 (PMC11377117; doi:10.1155/2024/4373987)
Supplement: Supplementary Materials — Graphs are presented in Figure S1 to S12. Table S1: principal component analysis for growth, seedling, morphophysiological, yield-related, and biochemical parameters under control and drought stress conditions. Table S2: correlation matrix (Pearson). [file 4373987.f1.zip › Supplementary Graphs.docx]

Figure. S1: Impact of drought stress on, germination percentage at 48 and 72 hours of all mutants in both (control and stress) conditions. Data presented as mean ± SE, mutants with different alphabets are statistically significantly different at P < 0.05.

Figure. S2: Impact of drought stress on, germination percentage at 96 and 120 hours of all mutants in both (control and stress) conditions. Data presented as mean ± SE, mutants with different alphabets are statistically significantly different at P < 0.05.

Figure. S3: Impact of drought stress on, germination rate and coefficient velocity of germination of all mutants in both (control and stress) conditions. Data presented as mean ± SE, mutants with different alphabets are statistically significantly different at P < 0.05.

Figure. S4: Impact of drought stress on, seed vigor and seedling height of all mutants in both (control and stress) conditions. Data presented as mean ± SE, mutants with different alphabets are statistically significantly different at P < 0.05.

Figure. S5: Impact of drought stress on, shoot and root length of all mutants in both (control and stress) conditions. Data presented as mean ± SE, mutants with different alphabets are statistically significantly different at P < 0.05.

Figure. S6: Impact of drought stress on, fresh and dry weight of all mutants in both (control and stress) conditions. Data presented as mean ± SE, mutants with different alphabets are statistically significantly different at P < 0.05.

Figure. S7: Impact of drought stress on, plant height and productive tillers of all mutants in both (control and stress) conditions. Data presented as mean ± SE, mutants with different alphabets are statistically significantly different at P < 0.05.

Figure. S8: Impact of drought stress on, panicle length and total spikelets of all mutants in both (control and stress) conditions. Data presented as mean ± SE, mutants with different alphabets are statistically significantly different at P < 0.05.

Figure. S9: Impact of drought stress on, empty spikelets and total weight of all mutants in both (control and stress) conditions. Data presented as mean ± SE, mutants with different alphabets are statistically significantly different at P < 0.05.

Figure. S10: Impact of drought stress on, fertility and yield of all mutants in both (control and stress) conditions. Data presented as mean ± SE, mutants with different alphabets are statistically significantly different at P < 0.05.

Figure. S11: Impact of drought stress on, chlorophyll and ascorbate peroxidase (APX) activity of all mutants in both (control and stress) conditions. Data presented as mean ± SE, mutants with different alphabets are statistically significantly different at P < 0.05.

Figure. S12: Impact of drought stress on catalase (CAT) and peroxidase (POD) of all mutants under both (control and stress) conditions. Data presented as mean ± SE, genotypes with different alphabets are statistically significantly different at P < 0.05.

**Table: S1:** Principal component analysis for growth, seedling, morpho-physiological, yield related and biochemical parameters under control and drought stress conditions.

| Eigenvalues: |  |  |  |  |  |  |  |  |  |  |  |  |  |  |  |  |  |
| --- | --- | --- | --- | --- | --- | --- | --- | --- | --- | --- | --- | --- | --- | --- | --- | --- | --- |
|  | F1 | F2 | F3 | F4 | F5 | F6 | F7 | F8 | F9 | F10 | F11 | F12 | F13 | F14 | F15 | F16 | F17 |
| Eigenvalue | 18.620 | 7.737 | 5.008 | 4.657 | 4.340 | 3.567 | 3.137 | 2.597 | 2.376 | 1.941 | 1.752 | 1.678 | 1.503 | 1.302 | 1.254 | 1.100 | 1.039 |
| Variability (%) | 25.861 | 10.745 | 6.955 | 6.468 | 6.028 | 4.954 | 4.357 | 3.607 | 3.300 | 2.696 | 2.433 | 2.330 | 2.088 | 1.809 | 1.741 | 1.528 | 1.443 |
| Cumulative % | 25.861 | 36.606 | 43.561 | 50.029 | 56.057 | 61.011 | 65.368 | 68.975 | 72.275 | 74.971 | 77.404 | 79.734 | 81.821 | 83.630 | 85.372 | 86.899 | 88.343 |

| Factor loadings: | |  |  |  |  |
| --- | --- | --- | --- | --- | --- |
|  | F1 | F2 | F3 | F4 | F5 |
| GP 48h-C | **0.720** | **0.309** | -0.111 | -0.138 | -0.221 |
| GP 48h-S | **0.713** | -0.147 | **0.141** | -0.404 | 0.135 |
| GP 48h-STI | **0.703** | -0.193 | **0.144** | -0.315 | 0.202 |
| GP 72h-C | **0.788** | **0.281** | -0.014 | 0.053 | -0.315 |
| GP 72h-S | **0.812** | **0.163** | -0.021 | -0.262 | 0.232 |
| GP 72h-STI | **0.549** | **0.088** | -0.088 | -0.353 | 0.514 |
| GP 96h-C | **0.738** | **0.299** | -0.047 | 0.136 | -0.408 |
| GP 96h-S | **0.875** | **0.195** | -0.152 | -0.117 | -0.067 |
| GP 96h-STI | **0.698** | **0.116** | -0.224 | -0.252 | 0.228 |
| GP 120h-C | **0.653** | **0.285** | -0.073 | 0.194 | -0.457 |
| GP 120h-S | **0.840** | **0.316** | -0.074 | 0.048 | -0.189 |
| GP 120h-STI | **0.758** | **0.296** | -0.092 | -0.082 | 0.089 |
| GR-C | **0.778** | **0.285** | -0.060 | -0.099 | -0.297 |
| GR-S | **0.828** | -0.014 | **0.045** | -0.297 | -0.022 |
| GR-STI | **0.098** | -0.363 | **0.160** | -0.264 | 0.339 |
| CVG%-C | **0.782** | **0.271** | -0.039 | 0.060 | -0.426 |
| CVG%-S | **0.925** | **0.100** | **0.022** | -0.193 | 0.022 |
| CVG%-STI | **0.586** | **-**0.012 | -0.039 | -0.307 | 0.499 |
| SV-C | **0.746** | -0.094 | -0.146 | 0.577 | -0.013 |
| SV-S | **0.886** | **0.141** | **0.014** | 0.345 | 0.126 |
| SV-STI | **0.687** | **0.476** | **0.112** | -0.107 | 0.221 |
| SH-C | **0.554** | -0.254 | -0.179 | 0.624 | 0.257 |
| SH-S | **0.787** | **0.127** | **0.024** | 0.390 | 0.318 |
| SH-STI | **0.505** | **0.497** | **0.183** | -0.186 | 0.167 |
| SL-C | **0.420** | -0.152 | -0.229 | 0.719 | 0.221 |
| SL-S | **0.646** | **0.257** | -0.114 | 0.426 | 0.371 |
| SL-STI | **0.480** | **0.513** | **0.039** | -0.128 | 0.272 |
| RL-C | **0.551** | -0.342 | -0.182 | 0.551 | 0.213 |
| RL-S | **0.762** | -0.040 | **0.054** | 0.388 | 0.217 |
| RL-STI | **0.354** | **0.429** | **0.260** | -0.230 | 0.070 |
| FW-C | **0.377** | -0.420 | -0.119 | 0.475 | -0.314 |
| FW-S | **0.407** | -0.364 | **0.043** | 0.308 | 0.032 |
| FW-STI | **0.009** | **0.219** | **0.154** | -0.079 | 0.481 |
| DW-C | **0.677** | -0.061 | -0.044 | 0.126 | -0.306 |
| DW-S | **0.643** | -0.197 | **0.048** | 0.294 | 0.234 |
| DW-STI | -0.114 | -0.203 | **0.083** | 0.000 | 0.523 |
| Chl-C | **0.228** | -0.441 | **0.204** | -0.038 | -0.057 |
| Chl-S | **0.207** | -0.503 | **0.038** | -0.069 | 0.110 |
| Chl-STI | **0.114** | -0.352 | -0.122 | -0.086 | 0.197 |
| APX-C | **0.429** | -0.505 | **0.573** | -0.110 | -0.141 |
| APX-S | **0.420** | -0.233 | **0.579** | -0.013 | -0.247 |
| APX-STI | -0.211 | 0.627 | -0.310 | 0.185 | -0.037 |
| CAT-C | **0.480** | -0.333 | **0.327** | -0.235 | -0.263 |
| CAT-S | **0.539** | -0.301 | **0.392** | -0.194 | -0.168 |
| CAT-STI | **0.182** | 0.130 | **0.219** | 0.054 | 0.157 |
| POD-C | **0.607** | -0.482 | **0.450** | -0.021 | -0.122 |
| POD-S | **0.533** | -0.525 | **0.481** | 0.015 | -0.204 |
| POD-STI | -0.125 | -0.238 | **0.107** | 0.182 | -0.240 |
| PH-C | -0.264 | -0.194 | **0.505** | 0.193 | -0.038 |
| PH-S | -0.002 | -0.397 | **0.395** | 0.054 | -0.242 |
| PH-STI | **0.311** | -0.163 | -0.216 | -0.176 | -0.203 |
| PT-C | -0.295 | **0.613** | **0.202** | 0.020 | 0.047 |
| PT-S | -0.349 | **0.586** | **0.220** | 0.153 | -0.026 |
| PT-STI | -0.124 | -0.007 | -0.024 | 0.260 | -0.088 |
| PL-C | -0.277 | 0.157 | **0.437** | 0.220 | -0.044 |
| PL-S | -0.049 | -0.110 | **0.443** | 0.096 | -0.093 |
| PL-STI | **0.259** | -0.298 | **0.005** | -0.127 | -0.051 |
| TS-C | **0.267** | -0.136 | **0.145** | -0.247 | -0.131 |
| TS-S | **0.312** | -0.300 | **0.176** | -0.220 | -0.080 |
| TS-STI | **0.136** | -0.251 | **0.102** | -0.026 | 0.035 |
| ES-C | **0.171** | -0.369 | -0.376 | -0.325 | 0.029 |
| ES-S | **0.351** | -0.125 | -0.363 | -0.504 | 0.026 |
| ES-STI | **0.255** | **0.345** | -0.164 | -0.215 | -0.077 |
| TW-C | -0.112 | **0.643** | **0.290** | -0.023 | -0.202 |
| TW-S | -0.180 | **0.274** | **0.425** | 0.004 | 0.338 |
| TW-STI | -0.098 | -0.448 | **0.101** | 0.002 | 0.470 |
| F-C | -0.328 | **0.295** | **0.440** | 0.100 | 0.293 |
| F-S | -0.170 | **0.432** | **0.580** | 0.198 | 0.235 |
| F-STI | **0.030** | **0.368** | **0.502** | 0.181 | 0.104 |
| Y-C | **0.027** | **0.534** | **0.401** | 0.013 | -0.317 |
| Y-S | **0.076** | **0.305** | **0.594** | 0.108 | 0.104 |
| Y-STI | **0.075** | -0.204 | **0.232** | 0.076 | 0.405 |

C= Control: S= Stress: STI= Stress Tolerance Index: GP 48h= Germination Percentage at 48 hours: GP 72h= Germination Percentage at 72 hours: GP 96h= Germination Percentage at 96 hours: GP 120h= Germination Percentage at 120 hours: GR= Germination rate: CVG= Coefficient Velocity of Germination: SV= Seed Vigor: S.H=Seedling Height: SL= Shoot Length: RL Root Length: FW= Fresh Weight: DW= Dry Weight: Chl= Chlorophyll Content: APX=Ascorbate peroxidase: CAT=Catalase: POD=Peroxidase: PH= Plant Height: PT= Productive Tillers: PL= Panicle Length: TS= Total Spikelets: ES= Empty Spikelets: TW= Total Weight: F= Fertility: Y= Yield
