# Supplementary material for: Development of Rice Mutants with Enhanced Resilience to Drought Stress and Their Evaluation by Lab Assay, Field, and Multivariate Analysis
Source: Scientifica (Cairo). 2024 Aug 29;2024:4373987. doi: 10.1155/2024/4373987 (PMC11377117; doi:10.1155/2024/4373987)
Supplement: Supplementary Materials — Graphs are presented in Figure S1 to S12. Table S1: principal component analysis for growth, seedling, morphophysiological, yield-related, and biochemical parameters under control and drought stress conditions. Table S2: correlation matrix (Pearson). [file 4373987.f1.zip › Supplementary Table 2.pdf]

Table S2: Correlation matrix (Pearson)

	Variables	GP 48h-C	GP 48h-S	GP 48h-STI	GP 72h-C	GP 72h-S	GP 72h-STI	GP 96h-C
1	GP 48h-C	**1**	**0.536**	**0.401**	**0.713**	**0.640**	**0.337**	**0.658**
2	GP 48h-S	**0.536**	**1**	**0.935**	**0.457**	**0.746**	**0.563**	**0.357**
3	GP 48h-STI	**0.401**	**0.935**	**1**	**0.445**	**0.721**	**0.558**	**0.348**
4	GP 72h-C	**0.713**	**0.457**	**0.445**	**1**	**0.694**	**0.282**	**0.869**
5	GP 72h-S	**0.640**	**0.746**	**0.721**	**0.694**	**1**	**0.815**	**0.524**
6	GP 72h-STI	**0.337**	**0.563**	**0.558**	**0.282**	**0.815**	**1**	0.181
7	GP 96h-C	**0.658**	**0.357**	**0.348**	**0.869**	**0.524**	0.181	**1**
8	GP 96h-S	**0.758**	**0.579**	**0.530**	**0.746**	**0.807**	**0.589**	**0.739**
9	GP 96h-STI	**0.562**	**0.519**	**0.474**	**0.442**	**0.762**	**0.766**	**0.349**
10	GP 120h-C	**0.580**	**0.274**	**0.253**	**0.800**	**0.416**	0.077	**0.967**
11	GP 120h-S	**0.662**	**0.430**	**0.407**	**0.842**	**0.679**	**0.429**	**0.863**
12	GP 120h-ST	**0.539**	**0.418**	**0.412**	**0.658**	**0.709**	**0.647**	**0.584**
13	GR-C	**0.963**	**0.565**	**0.450**	**0.790**	**0.659**	**0.314**	**0.752**
14	GR-S	**0.698**	**0.897**	**0.812**	**0.652**	**0.766**	**0.531**	**0.625**
15	GR-STI	**-0.294**	**0.375**	**0.418**	-0.101	0.163	**0.315**	-0.116
16	CVG%-C	**0.819**	**0.443**	**0.392**	**0.930**	**0.603**	0.184	**0.914**
17	CVG%-S	**0.753**	**0.790**	**0.734**	**0.756**	**0.910**	**0.644**	**0.678**
18	CVG%-STI	**0.278**	**0.595**	**0.595**	0.222	**0.741**	**0.918**	0.150
19	SV-C	**0.408**	**0.327**	**0.396**	**0.614**	**0.467**	0.223	**0.629**
20	SV-S	**0.586**	**0.461**	**0.488**	**0.673**	**0.630**	**0.393**	**0.670**
21	SV-STI	**0.556**	**0.394**	**0.361**	**0.508**	**0.596**	**0.518**	**0.514**
22	SH-C	0.169	0.203	**0.296**	**0.308**	**0.355**	**0.320**	**0.271**
23	SH-S	**0.447**	**0.410**	**0.465**	**0.524**	**0.564**	**0.402**	**0.495**
24	SH-STI	**0.450**	**0.351**	**0.320**	**0.407**	**0.433**	**0.263**	**0.398**
25	SL-C	0.189	0.044	0.101	**0.260**	0.225	0.159	0.210
26	SL-S	**0.460**	**0.240**	**0.247**	**0.480**	**0.502**	**0.342**	**0.436**
27	SL-STI	**0.444**	**0.280**	**0.245**	**0.412**	**0.472**	**0.332**	**0.392**
28	RL-C	0.130	**0.260**	**0.350**	**0.250**	**0.299**	**0.266**	**0.274**
29	RL-S	**0.387**	**0.440**	**0.519**	**0.463**	**0.464**	**0.315**	**0.470**
30	RL-STI	**0.359**	**0.252**	0.229	**0.315**	**0.293**	0.150	**0.286**
31	FW-C	0.135	0.133	**0.234**	**0.312**	0.056	-0.155	**0.355**
32	FW-S	**0.236**	0.225	**0.243**	0.211	0.149	-0.057	**0.232**
33	FW-STI	0.079	-0.004	-0.053	-0.087	0.109	0.144	-0.137
34	DW-C	**0.469**	**0.366**	**0.385**	**0.625**	**0.407**	0.108	**0.587**
35	DW-S	**0.276**	**0.436**	**0.476**	**0.401**	**0.432**	**0.270**	**0.401**
36	DW-STI	**-0.240**	0.066	0.077	**-0.288**	-0.013	0.172	**-0.285**
37	Chl-C	0.077	**0.305**	0.208	0.068	0.056	-0.058	0.119
38	Chl-S	0.037	**0.240**	0.204	0.062	0.063	-0.021	0.049
39	Chl-STI	0.007	0.094	0.119	0.044	0.049	0.012	-0.015
40	APX-C	0.131	**0.445**	**0.440**	0.198	0.214	0.095	0.134
41	APX-S	0.143	**0.292**	**0.277**	**0.312**	0.214	0.115	**0.266**
42	APX-STI	-0.039	**-0.410**	**-0.413**	0.064	-0.063	0.020	0.108
43	CAT-C	0.223	**0.404**	**0.413**	**0.351**	**0.296**	0.127	**0.288**
44	CAT-S	0.217	**0.446**	**0.459**	**0.392**	**0.366**	0.185	**0.300**

45	CAT-STI	0.052	0.087	0.089	0.170	0.209	0.169	0.104
46	POD-C	**0.272**	**0.553**	**0.545**	**0.318**	**0.379**	0.209	**0.306**
47	POD-S	0.191	**0.486**	**0.474**	**0.296**	**0.264**	0.086	**0.282**
48	POD-STI	-0.216	-0.140	-0.145	-0.069	**-0.294**	**-0.329**	-0.056
49	PH-C	**-0.301**	-0.156	-0.157	**-0.266**	**-0.279**	-0.191	-0.170
50	PH-S	-0.093	0.041	0.053	-0.037	-0.080	-0.063	-0.055
51	PH-STI	**0.266**	0.221	**0.234**	**0.284**	**0.251**	0.164	0.152
52	PT-C	-0.130	-0.229	-0.224	-0.056	-0.165	-0.208	-0.064
53	PT-S	-0.148	**-0.332**	**-0.342**	-0.130	**-0.323**	**-0.308**	-0.108
54	PT-STI	-0.018	-0.220	**-0.260**	-0.158	**-0.291**	-0.164	-0.100
55	PL-C	-0.096	**-0.257**	**-0.319**	-0.214	**-0.231**	-0.184	-0.088
56	PL-S	-0.051	-0.077	-0.099	-0.053	-0.020	-0.018	-0.021
57	PL-STI	0.047	0.196	**0.244**	0.181	**0.235**	0.187	0.075
58	TS-C	**0.240**	**0.240**	**0.255**	0.145	0.204	0.157	0.106
59	TS-S	0.152	**0.310**	**0.317**	0.080	0.159	0.147	0.041
60	TS-STI	-0.062	0.155	0.139	-0.051	-0.015	0.030	-0.059
61	ES-C	0.101	0.210	**0.234**	-0.005	0.156	0.215	-0.032
62	ES-S	**0.308**	**0.379**	**0.369**	0.156	**0.266**	**0.309**	0.180
63	ES-STI	**0.336**	0.180	0.124	**0.256**	0.191	0.166	**0.289**
64	TW-C	0.078	-0.089	-0.105	0.106	-0.056	-0.137	0.156
65	TW-S	-0.137	-0.061	0.004	-0.079	0.006	0.071	-0.126
66	TW-STI	**-0.250**	-0.002	0.084	-0.229	0.006	0.153	**-0.312**
67	F-C	**-0.243**	-0.165	-0.140	-0.226	-0.106	-0.037	**-0.284**
68	F-S	-0.127	-0.092	-0.058	-0.050	-0.023	-0.005	-0.097
69	F-STI	0.028	0.023	0.051	0.113	0.063	0.029	0.087
70	Y-C	0.183	-0.022	-0.058	**0.244**	0.086	-0.021	**0.235**
71	Y-S	0.104	0.046	0.099	0.176	0.103	0.025	0.101
72	Y-STI	-0.053	0.103	0.200	-0.055	0.023	0.030	-0.116

Values in bold are different from 0 with a significance level alpha=0.05

GP 96h-S	GP 96h-STI	GP 120h-C	GP 120h-S	GP 120h-STI	GR-C	GR-S	GR-STI	CVG%-C
**0.758**	**0.562**	**0.580**	**0.662**	**0.539**	**0.963**	**0.698**	**-0.294**	**0.819**
**0.579**	**0.519**	**0.274**	**0.430**	**0.418**	**0.565**	**0.897**	**0.375**	**0.443**
**0.530**	**0.474**	**0.253**	**0.407**	**0.412**	**0.450**	**0.812**	**0.418**	**0.392**
**0.746**	**0.442**	**0.800**	**0.842**	**0.658**	**0.790**	**0.652**	-0.101	**0.930**
**0.807**	**0.762**	**0.416**	**0.679**	**0.709**	**0.659**	**0.766**	0.163	**0.603**
**0.589**	**0.766**	0.077	**0.429**	**0.647**	**0.314**	**0.531**	**0.315**	0.184
**0.739**	**0.349**	**0.967**	**0.863**	**0.584**	**0.752**	**0.625**	-0.116	**0.914**
**1**	**0.872**	**0.658**	**0.889**	**0.836**	**0.796**	**0.753**	-0.007	**0.764**
**0.872**	**1**	**0.262**	**0.669**	**0.832**	**0.545**	**0.595**	0.129	**0.409**
**0.658**	**0.262**	**1**	**0.824**	**0.499**	**0.676**	**0.551**	-0.119	**0.866**
**0.889**	**0.669**	**0.824**	**1**	**0.888**	**0.739**	**0.656**	-0.028	**0.832**
**0.836**	**0.832**	**0.499**	**0.888**	**1**	**0.569**	**0.570**	0.116	**0.576**
**0.796**	**0.545**	**0.676**	**0.739**	**0.569**	**1**	**0.732**	**-0.316**	**0.893**
**0.753**	**0.595**	**0.551**	**0.656**	**0.570**	**0.732**	**1**	**0.359**	**0.673**
-0.007	0.129	-0.119	-0.028	0.116	**-0.316**	**0.359**	**1**	-0.214
**0.764**	**0.409**	**0.866**	**0.832**	**0.576**	**0.893**	**0.673**	-0.214	**1**
**0.914**	**0.772**	**0.591**	**0.810**	**0.748**	**0.799**	**0.883**	0.132	**0.752**
**0.624**	**0.826**	0.061	**0.460**	**0.699**	**0.266**	**0.568**	**0.426**	0.116
**0.573**	**0.368**	**0.617**	**0.643**	**0.498**	**0.475**	**0.457**	-0.015	**0.605**
**0.720**	**0.535**	**0.619**	**0.762**	**0.663**	**0.634**	**0.599**	-0.025	**0.679**
**0.655**	**0.585**	**0.447**	**0.677**	**0.695**	**0.577**	**0.511**	-0.050	**0.514**
**0.368**	**0.353**	**0.248**	**0.377**	**0.392**	0.211	**0.233**	0.031	**0.258**
**0.566**	**0.451**	**0.434**	**0.598**	**0.558**	**0.481**	**0.465**	-0.038	**0.496**
**0.427**	**0.305**	**0.338**	**0.463**	**0.412**	**0.458**	**0.391**	-0.116	**0.413**
**0.275**	**0.261**	0.212	**0.291**	**0.292**	0.191	0.124	-0.073	0.229
**0.495**	**0.402**	**0.399**	**0.527**	**0.489**	**0.463**	**0.350**	-0.140	**0.463**
**0.423**	**0.325**	**0.340**	**0.460**	**0.424**	**0.448**	**0.346**	-0.141	**0.409**
**0.340**	**0.297**	**0.256**	**0.351**	**0.334**	0.192	**0.283**	0.089	**0.242**
**0.497**	**0.366**	**0.418**	**0.530**	**0.466**	**0.431**	**0.477**	0.024	**0.460**
**0.294**	0.197	**0.239**	**0.322**	**0.278**	**0.351**	**0.277**	-0.107	**0.310**
**0.277**	0.107	**0.370**	**0.258**	0.088	0.198	**0.250**	0.105	**0.353**
**0.284**	0.154	0.193	0.205	0.088	**0.249**	**0.316**	0.136	**0.298**
-0.041	0.011	-0.207	-0.056	0.025	0.033	-0.047	-0.112	-0.062
**0.570**	**0.351**	**0.547**	**0.611**	**0.466**	**0.554**	**0.497**	-0.026	**0.636**
**0.434**	**0.307**	**0.338**	**0.446**	**0.396**	**0.301**	**0.480**	0.212	**0.351**
-0.196	-0.068	**-0.320**	**-0.254**	-0.123	**-0.297**	-0.067	0.216	**-0.349**
0.041	-0.075	0.145	0.063	-0.060	0.110	**0.287**	0.184	0.110
0.012	-0.060	0.026	-0.011	-0.073	0.041	0.193	0.199	0.068
-0.008	-0.024	-0.066	-0.060	-0.061	-0.018	0.046	0.124	0.021
0.184	0.104	0.081	0.172	0.144	0.213	**0.367**	**0.234**	0.200
**0.243**	0.145	**0.235**	**0.319**	**0.284**	**0.241**	**0.287**	0.126	**0.284**
0.010	0.034	0.160	0.121	0.130	-0.063	**-0.289**	**-0.285**	0.011
**0.307**	0.196	**0.241**	**0.299**	**0.234**	**0.291**	**0.396**	0.128	**0.325**
**0.344**	**0.240**	**0.247**	**0.343**	**0.300**	**0.309**	**0.431**	0.174	**0.341**

0.142	0.133	0.070	0.184	**0.237**	0.088	0.101	0.107	0.105
**0.370**	**0.250**	**0.251**	**0.316**	**0.248**	**0.343**	**0.526**	**0.260**	**0.325**
**0.278**	0.136	**0.242**	**0.262**	0.175	**0.275**	**0.464**	**0.265**	**0.297**
-0.223	**-0.289**	-0.017	-0.134	-0.192	-0.171	-0.122	0.059	-0.072
**-0.260**	**-0.278**	-0.109	-0.203	**-0.258**	**-0.273**	-0.184	0.097	-0.219
-0.028	-0.033	-0.049	-0.052	-0.073	-0.066	-0.007	0.103	-0.028
**0.282**	**0.297**	0.086	0.194	**0.238**	**0.263**	0.208	-0.022	**0.237**
-0.225	**-0.249**	-0.040	-0.092	-0.099	-0.118	-0.215	-0.124	-0.087
**-0.267**	**-0.266**	-0.079	-0.115	-0.093	-0.150	**-0.307**	-0.200	-0.138
-0.077	-0.014	-0.086	-0.058	0.005	-0.062	-0.190	-0.164	-0.115
**-0.237**	**-0.297**	-0.046	-0.159	**-0.231**	-0.096	**-0.241**	-0.186	-0.100
-0.023	-0.050	-0.048	-0.047	-0.065	-0.032	-0.116	-0.139	-0.005
**0.237**	**0.275**	0.001	0.128	0.190	0.070	0.138	0.060	0.105
0.184	0.153	0.085	0.159	0.153	0.224	0.208	0.016	0.222
0.191	0.212	0.023	0.128	0.158	0.160	**0.262**	0.112	0.143
0.072	0.145	-0.059	0.013	0.063	-0.026	0.129	0.129	-0.049
0.158	0.228	-0.029	0.066	0.103	0.094	0.149	0.085	0.045
**0.322**	**0.345**	0.147	**0.235**	**0.265**	**0.288**	**0.406**	0.189	0.227
**0.271**	0.229	**0.236**	**0.267**	**0.284**	**0.301**	**0.309**	0.033	**0.274**
-0.041	-0.140	0.150	0.113	0.075	0.085	-0.066	-0.174	0.118
-0.142	-0.100	-0.148	-0.106	-0.034	-0.141	-0.112	0.128	-0.122
-0.142	0.001	**-0.310**	**-0.260**	-0.165	**-0.257**	-0.082	**0.278**	**-0.267**
**-0.310**	**-0.237**	**-0.278**	**-0.274**	-0.207	**-0.294**	**-0.242**	0.077	**-0.306**
-0.153	-0.122	-0.109	-0.096	-0.030	-0.159	-0.135	0.053	-0.146
0.037	0.018	0.058	0.076	0.114	0.021	0.015	0.011	0.044
0.075	-0.049	0.194	0.206	0.172	0.206	0.022	-0.193	**0.233**
0.001	-0.071	0.056	0.101	0.105	0.139	0.036	-0.040	0.150
-0.058	-0.023	-0.118	-0.085	-0.059	-0.031	0.042	0.136	-0.054

CVG%-S	CVG%-STI	SV-C	SV-S	SV-STI	SH-C	SH-S	SH-STI	SL-C
**0.753**	**0.278**	**0.408**	**0.586**	**0.556**	0.169	**0.447**	**0.450**	0.189
**0.790**	**0.595**	**0.327**	**0.461**	**0.394**	0.203	**0.410**	**0.351**	0.044
**0.734**	**0.595**	**0.396**	**0.488**	**0.361**	**0.296**	**0.465**	**0.320**	0.101
**0.756**	0.222	**0.614**	**0.673**	**0.508**	**0.308**	**0.524**	**0.407**	**0.260**
**0.910**	**0.741**	**0.467**	**0.630**	**0.596**	**0.355**	**0.564**	**0.433**	0.225
**0.644**	**0.918**	0.223	**0.393**	**0.518**	**0.320**	**0.402**	**0.263**	0.159
**0.678**	0.150	**0.629**	**0.670**	**0.514**	**0.271**	**0.495**	**0.398**	0.210
**0.914**	**0.624**	**0.573**	**0.720**	**0.655**	**0.368**	**0.566**	**0.427**	**0.275**
**0.772**	**0.826**	**0.368**	**0.535**	**0.585**	**0.353**	**0.451**	**0.305**	**0.261**
**0.591**	0.061	**0.617**	**0.619**	**0.447**	**0.248**	**0.434**	**0.338**	0.212
**0.810**	**0.460**	**0.643**	**0.762**	**0.677**	**0.377**	**0.598**	**0.463**	**0.291**
**0.748**	**0.699**	**0.498**	**0.663**	**0.695**	**0.392**	**0.558**	**0.412**	**0.292**
**0.799**	**0.266**	**0.475**	**0.634**	**0.577**	0.211	**0.481**	**0.458**	0.191
**0.883**	**0.568**	**0.457**	**0.599**	**0.511**	**0.233**	**0.465**	**0.391**	0.124
0.132	**0.426**	-0.015	-0.025	-0.050	0.031	-0.038	-0.116	-0.073
**0.752**	0.116	**0.605**	**0.679**	**0.514**	**0.258**	**0.496**	**0.413**	0.229
**1**	**0.670**	**0.577**	**0.749**	**0.644**	**0.379**	**0.610**	**0.452**	**0.236**
**0.670**	**1**	**0.263**	**0.438**	**0.531**	**0.379**	**0.422**	0.223	0.184
**0.577**	**0.263**	**1**	**0.847**	**0.313**	**0.867**	**0.792**	0.148	**0.782**
**0.749**	**0.438**	**0.847**	**1**	**0.735**	**0.681**	**0.941**	**0.552**	**0.600**
**0.644**	**0.531**	**0.313**	**0.735**	**1**	0.174	**0.697**	**0.821**	0.141
**0.379**	**0.379**	**0.867**	**0.681**	0.174	**1**	**0.722**	-0.092	**0.887**
**0.610**	**0.422**	**0.792**	**0.941**	**0.697**	**0.722**	**1**	**0.602**	**0.639**
**0.452**	0.223	0.148	**0.552**	**0.821**	-0.092	**0.602**	**1**	-0.085
**0.236**	0.184	**0.782**	**0.600**	0.141	**0.887**	**0.639**	-0.085	**1**
**0.507**	**0.312**	**0.688**	**0.836**	**0.666**	**0.626**	**0.892**	**0.562**	**0.662**
**0.457**	**0.269**	0.189	**0.549**	**0.779**	0.008	**0.592**	**0.867**	-0.050
**0.367**	**0.350**	**0.850**	**0.661**	0.154	**0.934**	**0.686**	-0.069	**0.759**
**0.543**	**0.361**	**0.797**	**0.889**	**0.587**	**0.713**	**0.935**	**0.517**	**0.627**
**0.297**	0.103	-0.008	**0.360**	**0.665**	-0.220	**0.424**	**0.898**	-0.130
0.228	-0.060	**0.625**	**0.383**	-0.120	**0.509**	**0.291**	-0.180	**0.512**
**0.305**	0.037	**0.424**	**0.423**	0.098	**0.347**	**0.378**	0.099	**0.344**
0.035	0.084	-0.162	0.099	**0.286**	-0.084	0.205	**0.362**	-0.094
**0.580**	0.193	**0.574**	**0.596**	**0.354**	**0.361**	**0.467**	**0.248**	**0.260**
**0.521**	**0.369**	**0.629**	**0.679**	**0.372**	**0.565**	**0.655**	**0.247**	**0.466**
-0.119	0.158	-0.118	-0.057	-0.024	0.058	0.048	-0.036	0.062
0.170	-0.008	0.177	0.145	-0.005	0.071	0.116	0.065	0.015
0.117	-0.004	0.189	0.120	-0.070	0.163	0.136	-0.003	0.072
0.034	-0.008	0.116	0.055	-0.082	0.152	0.098	-0.035	0.070
**0.356**	0.212	0.193	**0.265**	0.120	0.181	0.230	0.082	0.010
**0.348**	0.219	**0.243**	**0.320**	**0.236**	0.202	**0.253**	0.109	0.038
-0.166	-0.066	-0.020	-0.047	0.098	-0.037	-0.059	0.003	0.045
**0.399**	0.187	0.199	**0.285**	0.226	0.079	0.216	0.198	0.007
**0.454**	**0.251**	**0.266**	**0.365**	**0.279**	0.160	**0.305**	**0.231**	0.059

0.181	0.184	0.126	0.194	0.192	0.147	0.196	0.113	0.099
**0.532**	**0.329**	**0.408**	**0.465**	0.230	**0.352**	**0.395**	0.115	0.175
**0.439**	0.222	**0.364**	**0.395**	0.166	**0.300**	**0.321**	0.078	0.153
-0.207	**-0.246**	-0.042	-0.102	-0.122	-0.036	-0.110	-0.093	0.041
**-0.243**	-0.187	-0.141	-0.193	-0.211	-0.116	-0.203	-0.162	-0.081
-0.021	-0.021	0.014	-0.089	-0.213	0.056	-0.120	**-0.231**	-0.041
**0.267**	0.200	0.181	0.138	0.038	0.191	0.117	-0.040	0.052
-0.215	-0.225	**-0.241**	-0.158	0.063	**-0.319**	-0.135	0.182	**-0.262**
**-0.315**	**-0.277**	**-0.234**	-0.166	0.046	**-0.262**	-0.141	0.107	-0.182
-0.203	-0.083	-0.003	-0.031	-0.024	0.098	-0.023	-0.137	0.170
-0.228	**-0.266**	-0.158	-0.125	-0.036	-0.177	-0.134	0.003	-0.099
-0.028	-0.069	-0.035	-0.032	-0.042	-0.020	-0.041	-0.053	-0.023
0.222	0.222	0.144	0.112	0.000	0.185	0.113	-0.062	0.096
**0.248**	0.110	0.111	0.184	0.198	0.023	0.113	0.152	-0.068
**0.244**	0.177	0.161	0.214	0.174	0.119	0.159	0.103	-0.002
0.064	0.135	0.084	0.090	0.039	0.137	0.094	-0.023	0.075
0.153	0.185	0.086	0.024	-0.037	0.093	-0.008	-0.033	-0.025
**0.310**	**0.274**	0.074	0.165	**0.260**	-0.009	0.123	**0.241**	-0.094
**0.235**	0.150	0.050	0.211	**0.371**	-0.049	0.175	**0.283**	-0.028
-0.041	-0.150	-0.118	-0.016	0.162	**-0.246**	-0.050	0.223	**-0.258**
-0.081	0.021	-0.147	-0.098	0.028	-0.103	-0.078	0.006	-0.094
-0.089	0.117	-0.057	-0.129	-0.189	0.114	-0.072	**-0.245**	0.128
-0.217	-0.070	**-0.242**	-0.181	-0.094	-0.175	-0.093	0.006	-0.155
-0.098	0.000	-0.128	-0.037	0.076	-0.105	0.034	0.108	-0.083
0.047	0.057	0.002	0.092	0.183	-0.021	0.123	0.156	-0.011
0.096	-0.056	-0.025	0.071	0.187	-0.139	0.001	0.170	-0.133
0.084	0.006	0.032	0.152	**0.232**	0.016	0.148	0.193	0.031
0.014	0.057	0.058	0.104	0.086	0.134	0.168	0.078	0.137

SL-S	SL-STI	RL-C	RL-S	RL-STI	FW-C	FW-S	FW-STI	DW-C
**0.460**	**0.444**	0.130	**0.387**	**0.359**	0.135	**0.236**	0.079	**0.469**
**0.240**	**0.280**	**0.260**	**0.440**	**0.252**	0.133	0.225	-0.004	**0.366**
**0.247**	**0.245**	**0.350**	**0.519**	0.229	**0.234**	**0.243**	-0.053	**0.385**
**0.480**	**0.412**	**0.250**	**0.463**	**0.315**	**0.312**	0.211	-0.087	**0.625**
**0.502**	**0.472**	**0.299**	**0.464**	**0.293**	0.056	0.149	0.109	**0.407**
**0.342**	**0.332**	**0.266**	**0.315**	0.150	-0.155	-0.057	0.144	0.108
**0.436**	**0.392**	**0.274**	**0.470**	**0.286**	**0.355**	**0.232**	-0.137	**0.587**
**0.495**	**0.423**	**0.340**	**0.497**	**0.294**	**0.277**	**0.284**	-0.041	**0.570**
**0.402**	**0.325**	**0.297**	**0.366**	0.197	0.107	0.154	0.011	**0.351**
**0.399**	**0.340**	**0.256**	**0.418**	**0.239**	**0.370**	0.193	-0.207	**0.547**
**0.527**	**0.460**	**0.351**	**0.530**	**0.322**	**0.258**	0.205	-0.056	**0.611**
**0.489**	**0.424**	**0.334**	**0.466**	**0.278**	0.088	0.088	0.025	**0.466**
**0.463**	**0.448**	0.192	**0.431**	**0.351**	0.198	**0.249**	0.033	**0.554**
**0.350**	**0.346**	**0.283**	**0.477**	**0.277**	**0.250**	**0.316**	-0.047	**0.497**
-0.140	-0.141	0.089	0.024	-0.107	0.105	0.136	-0.112	-0.026
**0.463**	**0.409**	**0.242**	**0.460**	**0.310**	**0.353**	**0.298**	-0.062	**0.636**
**0.507**	**0.457**	**0.367**	**0.543**	**0.297**	0.228	**0.305**	0.035	**0.580**
**0.312**	**0.269**	**0.350**	**0.361**	0.103	-0.060	0.037	0.084	0.193
**0.688**	0.189	**0.850**	**0.797**	-0.008	**0.625**	**0.424**	-0.162	**0.574**
**0.836**	**0.549**	**0.661**	**0.889**	**0.360**	**0.383**	**0.423**	0.099	**0.596**
**0.666**	**0.779**	0.154	**0.587**	**0.665**	-0.120	0.098	**0.286**	**0.354**
**0.626**	0.008	**0.934**	**0.713**	-0.220	**0.509**	**0.347**	-0.084	**0.361**
**0.892**	**0.592**	**0.686**	**0.935**	**0.424**	**0.291**	**0.378**	0.205	**0.467**
**0.562**	**0.867**	-0.069	**0.517**	**0.898**	-0.180	0.099	**0.362**	**0.248**
**0.662**	-0.050	**0.759**	**0.627**	-0.130	**0.512**	**0.344**	-0.094	**0.260**
**1**	**0.699**	**0.547**	**0.725**	**0.346**	0.183	**0.340**	**0.288**	**0.315**
**0.699**	**1**	0.021	**0.395**	**0.630**	**-0.234**	0.100	**0.444**	0.166
**0.547**	0.021	**1**	**0.740**	**-0.265**	**0.524**	**0.403**	-0.098	**0.395**
**0.725**	**0.395**	**0.740**	**1**	**0.405**	**0.414**	**0.427**	0.081	**0.497**
**0.346**	**0.630**	**-0.265**	**0.405**	**1**	-0.158	0.014	**0.240**	0.166
0.183	**-0.234**	**0.524**	**0.414**	-0.158	**1**	**0.598**	**-0.554**	**0.450**
**0.340**	0.100	**0.403**	**0.427**	0.014	**0.598**	**1**	**0.232**	**0.359**
**0.288**	**0.444**	-0.098	0.081	**0.240**	**-0.554**	**0.232**	**1**	-0.138
**0.315**	0.166	**0.395**	**0.497**	0.166	**0.450**	**0.359**	-0.138	**1**
**0.539**	**0.261**	**0.557**	**0.647**	0.108	**0.348**	**0.592**	0.210	**0.509**
0.075	0.025	0.021	0.032	-0.043	-0.208	0.158	**0.385**	**-0.515**
0.079	0.058	0.183	0.177	0.028	0.199	**0.276**	-0.061	0.084
0.140	0.086	**0.266**	0.166	-0.079	0.118	**0.319**	0.113	0.024
0.143	0.104	0.211	0.086	-0.120	-0.013	**0.239**	0.226	-0.032
-0.007	-0.038	0.203	**0.315**	0.129	0.226	**0.290**	-0.015	**0.359**
0.055	0.030	0.188	**0.287**	0.123	0.161	0.081	-0.101	**0.339**
0.102	0.121	-0.111	-0.176	-0.066	-0.215	**-0.440**	-0.097	-0.219
0.020	0.019	0.102	**0.302**	**0.251**	0.218	0.119	-0.146	**0.408**
0.092	0.068	0.169	**0.376**	**0.272**	0.200	0.114	-0.104	**0.428**

0.150	0.112	0.091	0.156	0.120	-0.094	-0.025	0.129	0.107
0.161	0.028	**0.375**	**0.464**	0.095	**0.358**	**0.410**	-0.059	**0.425**
0.098	-0.035	**0.329**	**0.407**	0.089	**0.398**	**0.405**	-0.112	**0.411**
-0.098	-0.162	-0.021	-0.050	-0.020	**0.234**	0.114	-0.154	0.004
-0.210	-0.202	-0.063	-0.130	-0.109	0.056	0.133	-0.069	**-0.284**
-0.224	**-0.250**	0.084	-0.047	-0.181	**0.260**	**0.263**	-0.158	0.022
0.020	-0.014	0.157	0.106	-0.050	0.189	0.092	-0.080	**0.374**
-0.071	0.155	**-0.338**	-0.204	0.191	**-0.290**	**-0.267**	0.125	-0.103
-0.066	0.093	**-0.295**	-0.218	0.100	**-0.298**	**-0.256**	0.124	-0.133
0.036	-0.095	0.068	-0.044	-0.170	-0.039	0.029	0.044	-0.085
-0.073	-0.018	-0.135	-0.126	-0.004	-0.205	-0.027	0.199	**-0.281**
-0.071	-0.089	-0.012	-0.014	-0.035	-0.063	0.054	0.161	-0.127
0.012	-0.077	0.145	0.132	-0.036	0.166	0.098	-0.049	0.177
0.039	0.143	0.134	0.158	0.059	0.058	0.195	0.024	0.147
0.017	0.048	**0.265**	**0.273**	0.041	0.119	0.187	-0.046	**0.245**
-0.026	-0.104	0.204	0.205	0.016	0.086	0.033	-0.083	0.182
-0.026	0.032	0.187	0.026	-0.104	0.158	0.173	-0.074	0.108
0.092	**0.252**	0.110	0.161	0.156	0.023	0.086	-0.021	0.181
0.144	0.224	-0.024	0.168	**0.234**	-0.153	-0.196	-0.024	0.102
-0.023	**0.241**	**-0.247**	-0.132	0.148	**-0.266**	-0.210	0.115	-0.169
-0.004	0.067	-0.138	-0.165	-0.017	-0.188	-0.108	0.150	**-0.242**
-0.037	-0.215	0.092	-0.052	-0.165	0.106	0.101	-0.009	-0.059
-0.109	-0.040	-0.224	-0.133	0.063	**-0.273**	**-0.237**	0.134	**-0.331**
0.005	0.064	-0.195	-0.027	0.142	-0.198	-0.161	0.126	-0.143
0.086	0.126	-0.108	0.071	0.164	-0.073	-0.024	0.093	0.062
-0.025	0.116	-0.190	-0.034	0.199	-0.171	-0.214	-0.029	-0.082
0.157	0.178	-0.050	0.089	0.217	-0.101	-0.089	0.109	-0.003
0.189	0.097	0.133	0.151	0.075	0.078	0.122	0.132	0.135

DW-S	DW-STI	Chl-C	Chl-S	Chl-STI	APX-C	APX-S	APX-STI	CAT-C
**0.276**	**-0.240**	0.077	0.037	0.007	0.131	0.143	-0.039	0.223
**0.436**	0.066	**0.305**	**0.240**	0.094	**0.445**	**0.292**	**-0.410**	**0.404**
**0.476**	0.077	0.208	0.204	0.119	**0.440**	**0.277**	**-0.413**	**0.413**
**0.401**	**-0.288**	0.068	0.062	0.044	0.198	**0.312**	0.064	**0.351**
**0.432**	-0.013	0.056	0.063	0.049	0.214	0.214	-0.063	**0.296**
**0.270**	0.172	-0.058	-0.021	0.012	0.095	0.115	0.020	0.127
**0.401**	**-0.285**	0.119	0.049	-0.015	0.134	**0.266**	0.108	**0.288**
**0.434**	-0.196	0.041	0.012	-0.008	0.184	**0.243**	0.010	**0.307**
**0.307**	-0.068	-0.075	-0.060	-0.024	0.104	0.145	0.034	0.196
**0.338**	**-0.320**	0.145	0.026	-0.066	0.081	**0.235**	0.160	**0.241**
**0.446**	**-0.254**	0.063	-0.011	-0.060	0.172	**0.319**	0.121	**0.299**
**0.396**	-0.123	-0.060	-0.073	-0.061	0.144	**0.284**	0.130	**0.234**
**0.301**	**-0.297**	0.110	0.041	-0.018	0.213	**0.241**	-0.063	**0.291**
**0.480**	-0.067	**0.287**	0.193	0.046	**0.367**	**0.287**	**-0.289**	**0.396**
0.212	0.216	0.184	0.199	0.124	**0.234**	0.126	**-0.285**	0.128
**0.351**	**-0.349**	0.110	0.068	0.021	0.200	**0.284**	0.011	**0.325**
**0.521**	-0.119	0.170	0.117	0.034	**0.356**	**0.348**	-0.166	**0.399**
**0.369**	0.158	-0.008	-0.004	-0.008	0.212	0.219	-0.066	0.187
**0.629**	-0.118	0.177	0.189	0.116	0.193	**0.243**	-0.020	0.199
**0.679**	-0.057	0.145	0.120	0.055	**0.265**	**0.320**	-0.047	**0.285**
**0.372**	-0.024	-0.005	-0.070	-0.082	0.120	**0.236**	0.098	0.226
**0.565**	0.058	0.071	0.163	0.152	0.181	0.202	-0.037	0.079
**0.655**	0.048	0.116	0.136	0.098	0.230	**0.253**	-0.059	0.216
**0.247**	-0.036	0.065	-0.003	-0.035	0.082	0.109	0.003	0.198
**0.466**	0.062	0.015	0.072	0.070	0.010	0.038	0.045	0.007
**0.539**	0.075	0.079	0.140	0.143	-0.007	0.055	0.102	0.020
**0.261**	0.025	0.058	0.086	0.104	-0.038	0.030	0.121	0.019
**0.557**	0.021	0.183	**0.266**	0.211	0.203	0.188	-0.111	0.102
**0.647**	0.032	0.177	0.166	0.086	**0.315**	**0.287**	-0.176	**0.302**
0.108	-0.043	0.028	-0.079	-0.120	0.129	0.123	-0.066	**0.251**
**0.348**	-0.208	0.199	0.118	-0.013	0.226	0.161	-0.215	0.218
**0.592**	0.158	**0.276**	**0.319**	**0.239**	**0.290**	0.081	**-0.440**	0.119
0.210	**0.385**	-0.061	0.113	0.226	-0.015	-0.101	-0.097	-0.146
**0.509**	**-0.515**	0.084	0.024	-0.032	**0.359**	**0.339**	-0.219	**0.408**
**1**	**0.410**	**0.237**	**0.245**	0.157	**0.324**	0.198	**-0.318**	**0.273**
**0.410**	**1**	0.110	0.207	0.208	-0.007	-0.144	-0.169	-0.107
**0.237**	0.110	**1**	**0.754**	**0.277**	**0.330**	0.214	**-0.321**	**0.270**
**0.245**	0.207	**0.754**	**1**	**0.836**	**0.272**	0.142	**-0.331**	0.223
0.157	0.208	**0.277**	**0.836**	**1**	0.108	0.019	-0.201	0.098
**0.324**	-0.007	**0.330**	**0.272**	0.108	**1**	**0.835**	**-0.726**	**0.676**
0.198	-0.144	0.214	0.142	0.019	**0.835**	**1**	**-0.246**	**0.660**
**-0.318**	-0.169	**-0.321**	**-0.331**	-0.201	**-0.726**	**-0.246**	**1**	**-0.361**
**0.273**	-0.107	**0.270**	0.223	0.098	**0.676**	**0.660**	**-0.361**	**1**
**0.291**	-0.115	**0.311**	**0.245**	0.094	**0.679**	**0.659**	**-0.360**	**0.893**

0.095	-0.017	0.044	0.005	-0.027	0.088	0.055	-0.056	-0.127
**0.506**	0.035	**0.395**	**0.297**	0.094	**0.866**	**0.730**	**-0.612**	**0.679**
**0.470**	0.040	**0.427**	**0.309**	0.079	**0.871**	**0.727**	**-0.637**	**0.671**
0.001	0.058	0.134	0.033	-0.088	0.110	0.090	-0.120	0.037
-0.053	0.228	0.206	0.050	-0.081	0.101	0.100	-0.076	-0.064
0.056	0.053	0.217	0.086	-0.048	**0.316**	**0.291**	**-0.232**	0.103
0.111	**-0.233**	-0.040	0.012	0.038	0.196	0.177	-0.139	0.181
-0.216	-0.162	**-0.246**	**-0.299**	**-0.235**	**-0.343**	-0.160	**0.389**	**-0.236**
-0.189	-0.118	**-0.302**	**-0.352**	**-0.262**	**-0.336**	-0.142	**0.396**	**-0.309**
0.052	0.099	-0.117	-0.108	-0.051	-0.082	-0.063	0.064	-0.213
-0.185	0.090	0.042	-0.067	-0.118	-0.060	0.042	0.117	-0.201
-0.087	0.050	0.136	0.082	0.008	0.179	0.224	-0.048	0.038
0.107	-0.053	0.098	0.155	0.130	**0.274**	0.211	-0.186	**0.267**
0.083	-0.005	0.149	0.204	0.192	0.142	0.111	-0.157	0.172
0.156	-0.042	**0.264**	**0.290**	0.205	**0.259**	0.223	-0.228	**0.231**
0.107	-0.082	0.177	0.152	0.053	0.226	0.228	-0.124	0.136
0.042	0.023	0.156	**0.275**	**0.285**	-0.029	-0.119	-0.137	0.042
0.113	-0.015	0.136	**0.282**	**0.316**	-0.031	-0.106	-0.109	0.149
0.056	-0.116	-0.115	-0.078	-0.023	-0.079	-0.001	0.165	0.103
-0.214	-0.130	-0.198	-0.179	-0.094	**-0.248**	-0.042	**0.365**	-0.212
-0.188	0.022	-0.157	-0.022	0.091	-0.121	-0.027	0.135	-0.187
-0.016	0.112	0.081	0.177	0.182	0.156	0.017	**-0.271**	0.057
-0.202	0.066	-0.068	-0.190	**-0.238**	-0.104	-0.047	0.166	-0.207
-0.047	0.013	-0.147	**-0.306**	**-0.337**	-0.037	0.092	0.206	-0.146
0.107	-0.014	-0.143	**-0.262**	**-0.267**	0.057	0.183	0.134	-0.031
-0.112	-0.072	-0.207	**-0.255**	-0.202	0.003	0.209	**0.238**	-0.050
-0.039	-0.080	-0.092	-0.062	-0.029	0.185	**0.265**	-0.029	0.031
0.070	-0.070	0.124	0.171	0.133	0.212	0.071	**-0.305**	0.109

CAT-S	CAT-STI	POD-C	POD-S	POD-STI	PH-C	PH-S	PH-STI	PT-C
0.217	0.052	**0.272**	0.191	-0.216	**-0.301**	-0.093	**0.266**	-0.130
**0.446**	0.087	**0.553**	**0.486**	-0.140	-0.156	0.041	0.221	-0.229
**0.459**	0.089	**0.545**	**0.474**	-0.145	-0.157	0.053	**0.234**	-0.224
**0.392**	0.170	**0.318**	**0.296**	-0.069	**-0.266**	-0.037	**0.284**	-0.056
**0.366**	0.209	**0.379**	**0.264**	**-0.294**	**-0.279**	-0.080	**0.251**	-0.165
0.185	0.169	0.209	0.086	**-0.329**	-0.191	-0.063	0.164	-0.208
**0.300**	0.104	**0.306**	**0.282**	-0.056	-0.170	-0.055	0.152	-0.064
**0.344**	0.142	**0.370**	**0.278**	-0.223	**-0.260**	-0.028	**0.282**	-0.225
**0.240**	0.133	**0.250**	0.136	**-0.289**	**-0.278**	-0.033	**0.297**	**-0.249**
**0.247**	0.070	**0.251**	**0.242**	-0.017	-0.109	-0.049	0.086	-0.040
**0.343**	0.184	**0.316**	**0.262**	-0.134	-0.203	-0.052	0.194	-0.092
**0.300**	**0.237**	**0.248**	0.175	-0.192	**-0.258**	-0.073	**0.238**	-0.099
**0.309**	0.088	**0.343**	**0.275**	-0.171	**-0.273**	-0.066	**0.263**	-0.118
**0.431**	0.101	**0.526**	**0.464**	-0.122	-0.184	-0.007	0.208	-0.215
0.174	0.107	**0.260**	**0.265**	0.059	0.097	0.103	-0.022	-0.124
**0.341**	0.105	**0.325**	**0.297**	-0.072	-0.219	-0.028	**0.237**	-0.087
**0.454**	0.181	**0.532**	**0.439**	-0.207	**-0.243**	-0.021	**0.267**	-0.215
**0.251**	0.184	**0.329**	0.222	**-0.246**	-0.187	-0.021	0.200	-0.225
**0.266**	0.126	**0.408**	**0.364**	-0.042	-0.141	0.014	0.181	**-0.241**
**0.365**	0.194	**0.465**	**0.395**	-0.102	-0.193	-0.089	0.138	-0.158
**0.279**	0.192	0.230	0.166	-0.122	-0.211	-0.213	0.038	0.063
0.160	0.147	**0.352**	**0.300**	-0.036	-0.116	0.056	0.191	**-0.319**
**0.305**	0.196	**0.395**	**0.321**	-0.110	-0.203	-0.120	0.117	-0.135
**0.231**	0.113	0.115	0.078	-0.093	-0.162	**-0.231**	-0.040	0.182
0.059	0.099	0.175	0.153	0.041	-0.081	-0.041	0.052	**-0.262**
0.092	0.150	0.161	0.098	-0.098	-0.210	-0.224	0.020	-0.071
0.068	0.112	0.028	-0.035	-0.162	-0.202	**-0.250**	-0.014	0.155
0.169	0.091	**0.375**	**0.329**	-0.021	-0.063	0.084	0.157	**-0.338**
**0.376**	0.156	**0.464**	**0.407**	-0.050	-0.130	-0.047	0.106	-0.204
**0.272**	0.120	0.095	0.089	-0.020	-0.109	-0.181	-0.050	0.191
0.200	-0.094	**0.358**	**0.398**	**0.234**	0.056	**0.260**	0.189	**-0.290**
0.114	-0.025	**0.410**	**0.405**	0.114	0.133	**0.263**	0.092	**-0.267**
-0.104	0.129	-0.059	-0.112	-0.154	-0.069	-0.158	-0.080	0.125
**0.428**	0.107	**0.425**	**0.411**	0.004	**-0.284**	0.022	**0.374**	-0.103
**0.291**	0.095	**0.506**	**0.470**	0.001	-0.053	0.056	0.111	-0.216
-0.115	-0.017	0.035	0.040	0.058	0.228	0.053	**-0.233**	-0.162
**0.311**	0.044	**0.395**	**0.427**	0.134	0.206	0.217	-0.040	**-0.246**
**0.245**	0.005	**0.297**	**0.309**	0.033	0.050	0.086	0.012	**-0.299**
0.094	-0.027	0.094	0.079	-0.088	-0.081	-0.048	0.038	**-0.235**
**0.679**	0.088	**0.866**	**0.871**	0.110	0.101	**0.316**	0.196	**-0.343**
**0.659**	0.055	**0.730**	**0.727**	0.090	0.100	**0.291**	0.177	-0.160
**-0.360**	-0.056	**-0.612**	**-0.637**	-0.120	-0.076	**-0.232**	-0.139	**0.389**
**0.893**	-0.127	**0.679**	**0.671**	0.037	-0.064	0.103	0.181	**-0.236**
**1**	**0.294**	**0.717**	**0.703**	0.017	-0.068	0.088	0.170	-0.216

**0.294**	**1**	0.135	0.136	-0.042	-0.035	-0.035	0.011	0.033
**0.717**	0.135	**1**	**0.951**	0.041	0.083	**0.282**	0.182	**-0.417**
**0.703**	0.136	**0.951**	**1**	**0.319**	0.101	**0.317**	0.193	**-0.394**
0.017	-0.042	0.041	**0.319**	**1**	0.085	0.182	0.071	-0.051
-0.068	-0.035	0.083	0.101	0.085	**1**	**0.590**	**-0.608**	-0.132
0.088	-0.035	**0.282**	**0.317**	0.182	**0.590**	**1**	**0.280**	-0.229
0.170	0.011	0.182	0.193	0.071	**-0.608**	**0.280**	**1**	-0.075
-0.216	0.033	**-0.417**	**-0.394**	-0.051	-0.132	-0.229	-0.075	**1**
**-0.320**	-0.028	**-0.447**	**-0.396**	0.021	-0.003	-0.078	-0.081	**0.863**
**-0.277**	-0.127	-0.125	-0.076	0.134	**0.236**	0.228	-0.058	**-0.255**
-0.214	-0.009	-0.054	-0.099	-0.085	**0.724**	**0.279**	**-0.579**	0.062
0.051	0.070	0.113	0.090	-0.051	**0.470**	**0.535**	-0.034	-0.103
**0.301**	0.100	0.200	0.221	0.038	**-0.283**	**0.275**	**0.598**	-0.194
0.157	0.002	0.212	0.208	0.034	0.227	**0.424**	0.141	-0.194
**0.263**	0.036	**0.284**	**0.276**	0.011	0.184	**0.412**	0.181	**-0.245**
0.204	0.062	0.172	0.161	-0.037	-0.006	0.075	0.082	-0.100
-0.001	-0.196	0.017	0.007	-0.018	-0.029	0.180	0.214	**-0.233**
0.109	-0.118	0.034	0.007	-0.111	-0.175	-0.063	0.141	-0.212
0.101	0.082	-0.033	-0.078	-0.190	**-0.339**	**-0.335**	0.066	0.014
-0.200	0.079	**-0.245**	**-0.241**	-0.106	0.091	0.017	-0.105	**0.531**
-0.157	0.058	-0.192	-0.194	-0.142	0.214	0.064	-0.203	**0.511**
0.070	-0.036	0.061	0.059	-0.020	0.140	0.087	-0.074	-0.075
-0.142	0.161	-0.171	-0.182	-0.053	0.185	0.017	-0.210	**0.372**
-0.052	**0.250**	-0.061	-0.072	-0.070	0.181	-0.005	-0.223	**0.459**
0.054	**0.236**	0.068	0.059	-0.062	0.132	-0.002	-0.159	**0.339**
-0.031	0.137	-0.022	-0.009	-0.009	0.141	0.088	-0.090	**0.312**
0.076	0.153	0.030	0.075	0.034	0.151	0.112	-0.081	**0.310**
0.131	0.010	0.062	0.106	0.069	-0.011	0.005	0.014	0.034

PT-S	PT-STI	PL-C	PL-S	PL-STI	TS-C	TS-S	TS-STI	ES-C
-0.148	-0.018	-0.096	-0.051	0.047	**0.240**	0.152	-0.062	0.101
**-0.332**	-0.220	**-0.257**	-0.077	0.196	**0.240**	**0.310**	0.155	0.210
**-0.342**	**-0.260**	**-0.319**	-0.099	**0.244**	**0.255**	**0.317**	0.139	**0.234**
-0.130	-0.158	-0.214	-0.053	0.181	0.145	0.080	-0.051	-0.005
**-0.323**	**-0.291**	**-0.231**	-0.020	**0.235**	0.204	0.159	-0.015	0.156
**-0.308**	-0.164	-0.184	-0.018	0.187	0.157	0.147	0.030	0.215
-0.108	-0.100	-0.088	-0.021	0.075	0.106	0.041	-0.059	-0.032
**-0.267**	-0.077	**-0.237**	-0.023	**0.237**	0.184	0.191	0.072	0.158
**-0.266**	-0.014	**-0.297**	-0.050	**0.275**	0.153	0.212	0.145	0.228
-0.079	-0.086	-0.046	-0.048	0.001	0.085	0.023	-0.059	-0.029
-0.115	-0.058	-0.159	-0.047	0.128	0.159	0.128	0.013	0.066
-0.093	0.005	**-0.231**	-0.065	0.190	0.153	0.158	0.063	0.103
-0.150	-0.062	-0.096	-0.032	0.070	0.224	0.160	-0.026	0.094
**-0.307**	-0.190	**-0.241**	-0.116	0.138	0.208	**0.262**	0.129	0.149
-0.200	-0.164	-0.186	-0.139	0.060	0.016	0.112	0.129	0.085
-0.138	-0.115	-0.100	-0.005	0.105	0.222	0.143	-0.049	0.045
**-0.315**	-0.203	-0.228	-0.028	0.222	**0.248**	**0.244**	0.064	0.153
**-0.277**	-0.083	**-0.266**	-0.069	0.222	0.110	0.177	0.135	0.185
**-0.234**	-0.003	-0.158	-0.035	0.144	0.111	0.161	0.084	0.086
-0.166	-0.031	-0.125	-0.032	0.112	0.184	0.214	0.090	0.024
0.046	-0.024	-0.036	-0.042	0.000	0.198	0.174	0.039	-0.037
**-0.262**	0.098	-0.177	-0.020	0.185	0.023	0.119	0.137	0.093
-0.141	-0.023	-0.134	-0.041	0.113	0.113	0.159	0.094	-0.008
0.107	-0.137	0.003	-0.053	-0.062	0.152	0.103	-0.023	-0.033
-0.182	0.170	-0.099	-0.023	0.096	-0.068	-0.002	0.075	-0.025
-0.066	0.036	-0.073	-0.071	0.012	0.039	0.017	-0.026	-0.026
0.093	-0.095	-0.018	-0.089	-0.077	0.143	0.048	-0.104	0.032
**-0.295**	0.068	-0.135	-0.012	0.145	0.134	**0.265**	0.204	0.187
-0.218	-0.044	-0.126	-0.014	0.132	0.158	**0.273**	0.205	0.026
0.100	-0.170	-0.004	-0.035	-0.036	0.059	0.041	0.016	-0.104
**-0.298**	-0.039	-0.205	-0.063	0.166	0.058	0.119	0.086	0.158
**-0.256**	0.029	-0.027	0.054	0.098	0.195	0.187	0.033	0.173
0.124	0.044	0.199	0.161	-0.049	0.024	-0.046	-0.083	-0.074
-0.133	-0.085	**-0.281**	-0.127	0.177	0.147	**0.245**	0.182	0.108
-0.189	0.052	-0.185	-0.087	0.107	0.083	0.156	0.107	0.042
-0.118	0.099	0.090	0.050	-0.053	-0.005	-0.042	-0.082	0.023
**-0.302**	-0.117	0.042	0.136	0.098	0.149	**0.264**	0.177	0.156
**-0.352**	-0.108	-0.067	0.082	0.155	0.204	**0.290**	0.152	**0.275**
**-0.262**	-0.051	-0.118	0.008	0.130	0.192	0.205	0.053	**0.285**
**-0.336**	-0.082	-0.060	0.179	**0.274**	0.142	**0.259**	0.226	-0.029
-0.142	-0.063	0.042	0.224	0.211	0.111	0.223	0.228	-0.119
**0.396**	0.064	0.117	-0.048	-0.186	-0.157	-0.228	-0.124	-0.137
**-0.309**	-0.213	-0.201	0.038	**0.267**	0.172	**0.231**	0.136	0.042
**-0.320**	**-0.277**	-0.214	0.051	**0.301**	0.157	**0.263**	0.204	-0.001

-0.028	-0.127	-0.009	0.070	0.100	0.002	0.036	0.062	-0.196
**-0.447**	-0.125	-0.054	0.113	0.200	0.212	**0.284**	0.172	0.017
**-0.396**	-0.076	-0.099	0.090	0.221	0.208	**0.276**	0.161	0.007
0.021	0.134	-0.085	-0.051	0.038	0.034	0.011	-0.037	-0.018
-0.003	**0.236**	**0.724**	**0.470**	**-0.283**	0.227	0.184	-0.006	-0.029
-0.078	0.228	**0.279**	**0.535**	**0.275**	**0.424**	**0.412**	0.075	0.180
-0.081	-0.058	**-0.579**	-0.034	**0.598**	0.141	0.181	0.082	0.214
**0.863**	**-0.255**	0.062	-0.103	-0.194	-0.194	**-0.245**	-0.100	**-0.233**
**1**	**0.257**	0.149	0.059	-0.118	-0.167	-0.143	0.033	**-0.237**
**0.257**	**1**	0.171	**0.268**	0.095	0.041	0.190	**0.261**	-0.004
0.149	0.171	**1**	**0.577**	**-0.475**	0.166	0.037	-0.150	-0.184
0.059	**0.268**	**0.577**	**1**	**0.443**	0.197	**0.303**	**0.232**	-0.049
-0.118	0.095	**-0.475**	**0.443**	**1**	0.033	**0.281**	**0.404**	0.142
-0.167	0.041	0.166	0.197	0.033	**1**	**0.778**	-0.125	**0.510**
-0.143	0.190	0.037	**0.303**	**0.281**	**0.778**	**1**	**0.518**	**0.467**
0.033	**0.261**	-0.150	**0.232**	**0.404**	-0.125	**0.518**	**1**	0.037
**-0.237**	-0.004	-0.184	-0.049	0.142	**0.510**	**0.467**	0.037	**1**
**-0.241**	-0.037	**-0.286**	**-0.244**	0.038	**0.547**	**0.552**	0.123	**0.702**
0.045	0.074	-0.217	-0.201	0.011	-0.027	0.106	0.219	**-0.301**
**0.589**	0.109	**0.248**	0.105	-0.168	0.196	0.034	-0.189	-0.159
**0.472**	-0.076	**0.240**	0.165	-0.091	0.077	-0.008	-0.098	0.036
-0.177	-0.198	-0.013	0.076	0.101	-0.078	0.000	0.102	0.226
**0.259**	-0.194	**0.237**	0.031	**-0.231**	-0.190	**-0.234**	-0.102	**-0.551**
**0.402**	-0.074	0.205	0.058	-0.154	-0.175	-0.173	-0.024	**-0.646**
**0.350**	0.049	0.110	0.070	-0.029	-0.065	-0.025	0.061	**-0.483**
**0.405**	0.156	**0.283**	0.188	-0.118	0.225	0.096	-0.126	**-0.250**
**0.384**	0.110	0.204	0.217	0.003	0.123	0.090	-0.013	-0.177
0.012	-0.053	-0.099	0.006	0.115	-0.058	0.029	0.104	0.075

ES-S	ES-STI	TW-C	TW-S	TW-STI	F-C	F-S	F-STI	Y-C
**0.308**	**0.336**	0.078	-0.137	**-0.250**	**-0.243**	-0.127	0.028	0.183
**0.379**	0.180	-0.089	-0.061	-0.002	-0.165	-0.092	0.023	-0.022
**0.369**	0.124	-0.105	0.004	0.084	-0.140	-0.058	0.051	-0.058
0.156	**0.256**	0.106	-0.079	-0.229	-0.226	-0.050	0.113	**0.244**
**0.266**	0.191	-0.056	0.006	0.006	-0.106	-0.023	0.063	0.086
**0.309**	0.166	-0.137	0.071	0.153	-0.037	-0.005	0.029	-0.021
0.180	**0.289**	0.156	-0.126	**-0.312**	**-0.284**	-0.097	0.087	**0.235**
**0.322**	**0.271**	-0.041	-0.142	-0.142	**-0.310**	-0.153	0.037	0.075
**0.345**	0.229	-0.140	-0.100	0.001	**-0.237**	-0.122	0.018	-0.049
0.147	**0.236**	0.150	-0.148	**-0.310**	**-0.278**	-0.109	0.058	0.194
**0.235**	**0.267**	0.113	-0.106	**-0.260**	**-0.274**	-0.096	0.076	0.206
**0.265**	**0.284**	0.075	-0.034	-0.165	-0.207	-0.030	0.114	0.172
**0.288**	**0.301**	0.085	-0.141	**-0.257**	**-0.294**	-0.159	0.021	0.206
**0.406**	**0.309**	-0.066	-0.112	-0.082	**-0.242**	-0.135	0.015	0.022
0.189	0.033	-0.174	0.128	**0.278**	0.077	0.053	0.011	-0.193
0.227	**0.274**	0.118	-0.122	**-0.267**	**-0.306**	-0.146	0.044	**0.233**
**0.310**	**0.235**	-0.041	-0.081	-0.089	-0.217	-0.098	0.047	0.096
**0.274**	0.150	-0.150	0.021	0.117	-0.070	0.000	0.057	-0.056
0.074	0.050	-0.118	-0.147	-0.057	**-0.242**	-0.128	0.002	-0.025
0.165	0.211	-0.016	-0.098	-0.129	-0.181	-0.037	0.092	0.071
**0.260**	**0.371**	0.162	0.028	-0.189	-0.094	0.076	0.183	0.187
-0.009	-0.049	**-0.246**	-0.103	0.114	-0.175	-0.105	-0.021	-0.139
0.123	0.175	-0.050	-0.078	-0.072	-0.093	0.034	0.123	0.001
**0.241**	**0.283**	0.223	0.006	**-0.245**	0.006	0.108	0.156	0.170
-0.094	-0.028	**-0.258**	-0.094	0.128	-0.155	-0.083	-0.011	-0.133
0.092	0.144	-0.023	-0.004	-0.037	-0.109	0.005	0.086	-0.025
**0.252**	0.224	**0.241**	0.067	-0.215	-0.040	0.064	0.126	0.116
0.110	-0.024	**-0.247**	-0.138	0.092	-0.224	-0.195	-0.108	-0.190
0.161	0.168	-0.132	-0.165	-0.052	-0.133	-0.027	0.071	-0.034
0.156	**0.234**	0.148	-0.017	-0.165	0.063	0.142	0.164	0.199
0.023	-0.153	**-0.266**	-0.188	0.106	**-0.273**	-0.198	-0.073	-0.171
0.086	-0.196	-0.210	-0.108	0.101	**-0.237**	-0.161	-0.024	-0.214
-0.021	-0.024	0.115	0.150	-0.009	0.134	0.126	0.093	-0.029
0.181	0.102	-0.169	**-0.242**	-0.059	**-0.331**	-0.143	0.062	-0.082
0.113	0.056	-0.214	-0.188	-0.016	-0.202	-0.047	0.107	-0.112
-0.015	-0.116	-0.130	0.022	0.112	0.066	0.013	-0.014	-0.072
0.136	-0.115	-0.198	-0.157	0.081	-0.068	-0.147	-0.143	-0.207
**0.282**	-0.078	-0.179	-0.022	0.177	-0.190	**-0.306**	**-0.262**	**-0.255**
**0.316**	-0.023	-0.094	0.091	0.182	**-0.238**	**-0.337**	**-0.267**	-0.202
-0.031	-0.079	**-0.248**	-0.121	0.156	-0.104	-0.037	0.057	0.003
-0.106	-0.001	-0.042	-0.027	0.017	-0.047	0.092	0.183	0.209
-0.109	0.165	**0.365**	0.135	**-0.271**	0.166	0.206	0.134	**0.238**
0.149	0.103	-0.212	-0.187	0.057	-0.207	-0.146	-0.031	-0.050
0.109	0.101	-0.200	-0.157	0.070	-0.142	-0.052	0.054	-0.031

-0.118	0.082	0.079	0.058	-0.036	0.161	**0.250**	**0.236**	0.137
0.034	-0.033	**-0.245**	-0.192	0.061	-0.171	-0.061	0.068	-0.022
0.007	-0.078	**-0.241**	-0.194	0.059	-0.182	-0.072	0.059	-0.009
-0.111	-0.190	-0.106	-0.142	-0.020	-0.053	-0.070	-0.062	-0.009
-0.175	**-0.339**	0.091	0.214	0.140	0.185	0.181	0.132	0.141
-0.063	**-0.335**	0.017	0.064	0.087	0.017	-0.005	-0.002	0.088
0.141	0.066	-0.105	-0.203	-0.074	-0.210	-0.223	-0.159	-0.090
-0.212	0.014	**0.531**	**0.511**	-0.075	**0.372**	**0.459**	**0.339**	**0.312**
**-0.241**	0.045	**0.589**	**0.472**	-0.177	**0.259**	**0.402**	**0.350**	**0.405**
-0.037	0.074	0.109	-0.076	-0.198	-0.194	-0.074	0.049	0.156
**-0.286**	-0.217	**0.248**	**0.240**	-0.013	**0.237**	0.205	0.110	**0.283**
**-0.244**	-0.201	0.105	0.165	0.076	0.031	0.058	0.070	0.188
0.038	0.011	-0.168	-0.091	0.101	**-0.231**	-0.154	-0.029	-0.118
**0.547**	-0.027	0.196	0.077	-0.078	-0.190	-0.175	-0.065	0.225
**0.552**	0.106	0.034	-0.008	0.000	**-0.234**	-0.173	-0.025	0.096
0.123	0.219	-0.189	-0.098	0.102	-0.102	-0.024	0.061	-0.126
**0.702**	**-0.301**	-0.159	0.036	0.226	**-0.551**	**-0.646**	**-0.483**	**-0.250**
**1**	**0.339**	-0.044	-0.084	-0.016	**-0.476**	**-0.507**	**-0.322**	-0.146
**0.339**	**1**	0.146	-0.219	**-0.385**	0.059	0.023	-0.005	0.140
-0.044	0.146	**1**	**0.461**	**-0.603**	0.215	**0.328**	**0.290**	**0.769**
-0.084	-0.219	**0.461**	**1**	**0.400**	**0.339**	**0.396**	**0.287**	**0.283**
-0.016	**-0.385**	**-0.603**	**0.400**	**1**	0.094	0.001	-0.070	**-0.555**
**-0.476**	0.059	0.215	**0.339**	0.094	**1**	**0.740**	**0.278**	0.185
**-0.507**	0.023	**0.328**	**0.396**	0.001	**0.740**	**1**	**0.849**	**0.277**
**-0.322**	-0.005	**0.290**	**0.287**	-0.070	**0.278**	**0.849**	**1**	**0.255**
-0.146	0.140	**0.769**	**0.283**	**-0.555**	0.185	**0.277**	**0.255**	**1**
-0.159	-0.045	**0.456**	**0.716**	0.160	**0.301**	**0.352**	**0.267**	**0.562**
0.017	-0.178	**-0.286**	**0.450**	**0.716**	0.127	0.094	0.032	**-0.411**

Y-S	Y-STI
0.104	-0.053
0.046	0.103
0.099	0.200
0.176	-0.055
0.103	0.023
0.025	0.030
0.101	-0.116
0.001	-0.058
-0.071	-0.023
0.056	-0.118
0.101	-0.085
0.105	-0.059
0.139	-0.031
0.036	0.042
-0.040	0.136
0.150	-0.054
0.084	0.014
0.006	0.057
0.032	0.058
0.152	0.104
**0.232**	0.086
0.016	0.134
0.148	0.168
0.193	0.078
0.031	0.137
0.157	0.189
0.178	0.097
-0.050	0.133
0.089	0.151
0.217	0.075
-0.101	0.078
-0.089	0.122
0.109	0.132
-0.003	0.135
-0.039	0.070
-0.080	-0.070
-0.092	0.124
-0.062	0.171
-0.029	0.133
0.185	0.212
**0.265**	0.071
-0.029	**-0.305**
0.031	0.109
0.076	0.131

0.153	0.010
0.030	0.062
0.075	0.106
0.034	0.069
0.151	-0.011
0.112	0.005
-0.081	0.014
**0.310**	0.034
**0.384**	0.012
0.110	-0.053
0.204	-0.099
0.217	0.006
0.003	0.115
0.123	-0.058
0.090	0.029
-0.013	0.104
-0.177	0.075
-0.159	0.017
-0.045	-0.178
**0.456**	**-0.286**
**0.716**	**0.450**
0.160	**0.716**
**0.301**	0.127
**0.352**	0.094
**0.267**	0.032
**0.562**	**-0.411**
**1**	**0.510**
**0.510**	**1**
